# Primary Liver Cancer Trends Worldwide and in China: Analysis of GLOBOCAN 2022 Data and Disease Management Implications

**DOI:** 10.1002/poh2.70033

**Published:** 2026-03-27

**Authors:** Jiayan Yan, Jiayi Wang, Jian Fan, Xinyi Cui, Yuxi Zhang, Xinrong Yang, Qiang Gao, Zhenbin Ding, Zhaoyou Tang, Jia Fan, Dan G. Duda, Ao Huang, Jian Zhou

**Affiliations:** ^1^ Department of Hepatobiliary Surgery and Liver Transplantation, Liver Cancer Institute, Zhongshan Hospital Fudan University; Key Laboratory of Carcinogenesis and Cancer Invasion, Ministry of Education Shanghai China; ^2^ Department of Nephrology, Zhongshan Hospital Fudan University Shanghai China; ^3^ Department of General Practice The First Affiliated Hospital of Naval Medical University, Changhai Hospital Shanghai China; ^4^ Steele Laboratories for Tumor Biology, Department of Radiation Oncology Massachusetts General Hospital and Harvard Medical School Boston Massachusetts USA; ^5^ Transplant Oncology and Therapeutics Program, Department of Surgery Houston Methodist Academic Institute Houston Texas USA

## Abstract

Primary liver cancer remains a significant global public health challenge, characterized by persistently high incidence and mortality. This review synthesizes current epidemiological data to analyze trends and etiological shifts, with particular emphasis on China, which bears over 40% of the global burden. Findings highlight a transition in dominant risk factors from viral hepatitis to metabolic dysfunction‐associated steatotic liver disease (MASLD), alongside persistent threats from aflatoxin exposure and lifestyle behaviors. Evidence‐based prevention strategies, including universal hepatitis B virus (HBV) vaccination, antiviral therapy expansion, aflatoxin control, and early metabolic intervention, are critical to reducing disease burden. The integration of artificial intelligence into screening and management represents a promising advancement. A multi‐faceted approach combining vaccination, surveillance, lifestyle modification, and technological innovation is essential for effective global liver cancer control.

## Graphical abstract

Primary liver cancer remains a major global health burden, with China accounting for over 40% of cases. The predominant etiology is shifting from viral hepatitis to metabolic dysfunction‐associated steatotic liver disease (MASLD), while aflatoxin exposure and unhealthy lifestyles remain important contributors. Comprehensive control strategies—including HBV vaccination, antiviral therapy, metabolic risk management, and AI‐assisted screening—are essential to reduce incidence and mortality.

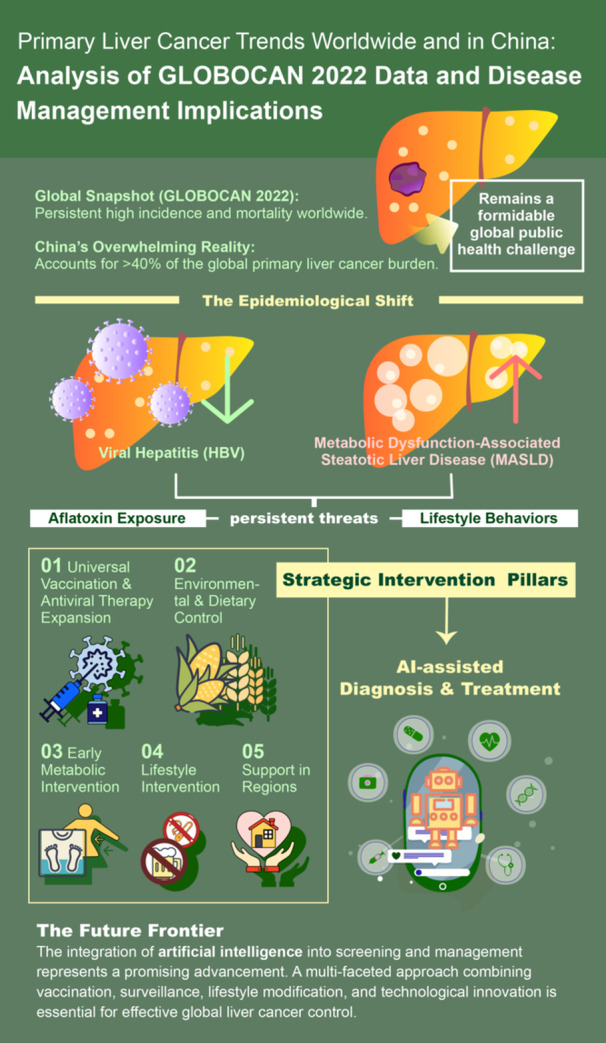

## 1 Introduction

Primary liver cancer (hereafter referred to as liver cancer) is a common malignant tumor worldwide and represents a major public health concern. Despite advancements in medical diagnosis, treatment modalities, and preventive strategies over recent decades, both the incidence and mortality of liver cancer remain persistently high. The situation highlights an urgent need for comprehensive epidemiological exploration and targeted interventions [1, 2, 3, 4, 5].

The most recent global cancer burden estimates, derived from the *GLOBOCAN* 2022 database of the International Agency for Research on Cancer (IARC), offer valuable insights into current liver cancer epidemiology. These data highlight significant trends in both incidence and mortality rates while shedding light on the factors driving these changes [2]. Globally, liver cancer ranked sixth in terms of newly diagnosed cases and third in cancer‐related mortality in 2022 [2]. Approximately 0.87 million new cases were reported worldwide, with hepatocellular carcinoma (HCC) accounting for nearly 80.0% of these cases. Particularly, projections suggest that if current trends continue unchecked, the annual number of new liver cancer cases could rise to 1.52 million by 2050, with associated deaths reaching 1.37 million [6].

China bears the heaviest burden of liver cancer globally, displaying both shared features with global patterns and region‐specific epidemiological characteristics. In 2022, liver cancer ranked fourth in incidence and second in mortality among all cancer types in China, following only lung cancer [7, 8]. Remarkably, China accounts for over 40% of global liver cancer cases and deaths [9]. Therefore, special attention will be paid to the epidemiological trends of primary liver cancer in China. The high global incidence of liver cancer is associated with several well‐defined risk factors such as hepatitis B virus (HBV), hepatitis C virus (HCV), aflatoxin exposure, and alcohol consumption, and metabolic dysfunction‐associated steatohepatitis (MASH) [10, 11, 12, 13, 14, 15, 16]. These etiological factors also play a crucial role in the pathogenesis of primary liver cancer in China; however, HBV‐related liver cancer and metabolic dysfunction‐associated steatotic liver disease (MASLD)‐related liver cancer have emerged as major etiologies of the disease in recent years [17, 18, 19].

This review provides a systematic synthesis of recent epidemiological data on primary liver cancer at both global and national (Chinese) levels mainly based on GLOBOCAN 2022 data and Global Burden of Disease (GBD) 2021 data. GLOBOCAN 2022 data are primarily derived from national registry data. Where data are missing or outdated, estimates are extrapolated based on cancer incidence patterns or mortality‐to‐incidence ratios from neighboring countries or those with similar socioeconomic development levels [2]. The GBD data are a system centered on Bayesian spatiotemporal regression. It integrates multisource data, including global death registries, disease surveillance, medical records, surveys, and literature, and incorporates covariates such as social development, healthcare accessibility, and behavioral risk factors for standardization. The model employs the Cause of Death Ensemble model to correct cause‐of‐death data and utilizes Disease Modelling Meta‐Regression (DisMod‐MR) to ensure epidemiological consistency among incidence, prevalence, and mortality rates, ultimately generating continuous estimates of disease burden from 1990 to the present [13]. The review focuses on geographical disparities and temporal trends in incidence and mortality. By examining shifts in major risk factors and their epidemiological characteristics, it aims to elucidate the key mechanisms driving changes in liver cancer burden. Finally, it identifies current gaps in prevention and control strategies and proposes future directions for optimizing intervention policies and diagnostic technologies. These efforts are intended to support the development of robust, evidence‐based strategies for liver cancer prevention and control in China and worldwide.

## 2 Evolving Epidemiology of Liver Cancer

Pathologically, liver cancer comprises three principal subtypes: HCC, intrahepatic cholangiocarcinoma (ICC), and combined HCC‐ICC. These subtypes differ in epidemiological characteristics, clinicopathological features, molecular alterations, and clinical management [5]. Among them, HCC is the most prevalent, with key etiological factors including chronic infection with HBV or HCV, alcohol‐associated liver disease, and MASH [6].

Liver cancer poses a significant burden on public health, severely impacting survival and quality of life in both China and globally. According to the National Cancer Center of China, approximately 367,700 new cases were diagnosed in 2022, accounting for 7.6% of all newly diagnosed cancers nationwide [14]. Liver cancer ranks as the fourth most common malignancy in China, following lung, colorectal, and thyroid cancers. It is also the second leading cause of cancer‐related deaths, with an estimated 400,415 deaths in 2022, accounting for more than 12.3% of global cancer mortality burden. The 5‐year survival rate for liver cancer in China remains low, at just 14.1% [20]. Globally, liver cancer ranks as the sixth most commonly diagnosed cancer and the third leading cause of cancer‐related death, according to GLOBOCAN 2022. The high fatality is primarily attributable to diagnosis at advanced stages and limited curative treatment options. A recent study published in *The Lancet* projected that without effective intervention, the annual number of new liver cancer cases will increase by approximately 76.0%, from 870,000 in 2022 to 1.52 million by 2050. During the same period, global liver cancer deaths are expected to rise by 80.3%, from 760,000 to 1.37 million, with the steepest increases anticipated in African regions. Importantly, an estimated 60% of liver cancer cases are preventable through the control of modifiable risk factors such as HBV, HCV, MASH, and alcohol consumption [6].

## 3 Incidence Trends of Liver Cancers Globally and in China

### 3.1 New Cases and Incidence Worldwide

**Figure 1:**
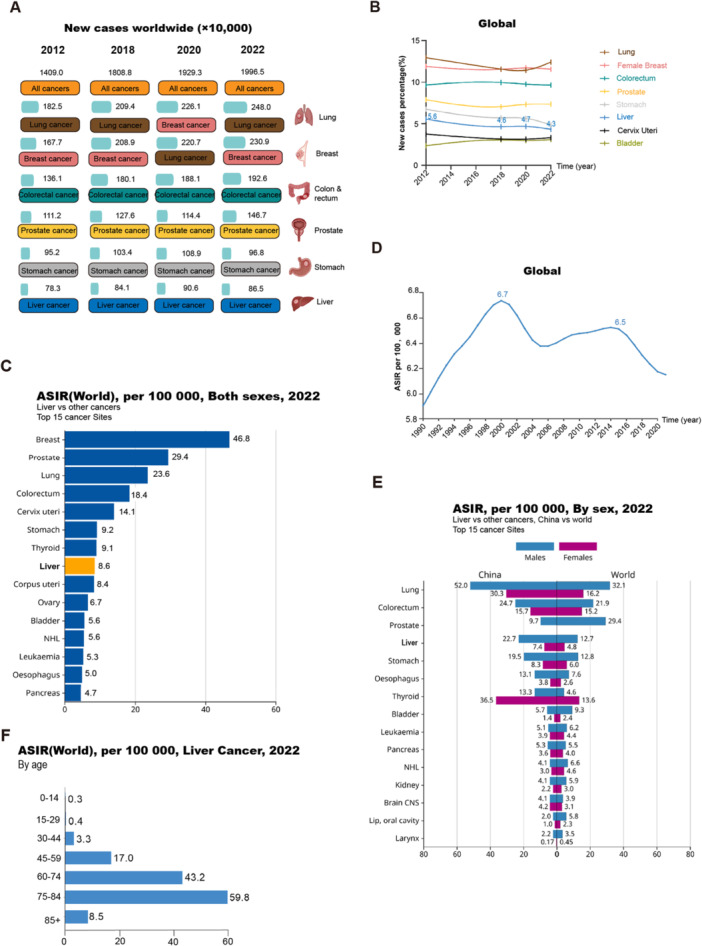
Global new cases and temporal trends of the most frequent cancers. (A) Global cancer incidence (*10,000 cases) in 2012, 2018, 2020, and 2022, based on GLOBOCAN data. (B) Proportion of liver cancer among all newly diagnosed cancers globally in 2012, 2018, 2020, and 2022 based on GLOBOCAN data; no data were available for 2014 and 2016. (C) The 15 most prevalent cancers by age‐standardized incidence rate (ASIR) in both sexes globally in 2022, with liver cancer highlighted for comparison based on GLOBOCAN 2022 data. (D) Temporal trends in global liver cancer ASIR from 1990 to 2021, based on GBD data. (E) ASIR rankings of the top 15 cancers in China compared with the global population, stratified by sex based on GLOBOCAN 2022 data. (F) Global age‐specific ASIR distribution of liver cancer based on GLOBOCAN 2022 data. (C) and (E) share the same map source: World Health Organization (https://gco.iarc.who.int/today/). CNS, central nervous system; GBD, Global Burden of Disease; NHL, non‐Hodgkin lymphoma.

According to GLOBOCAN 2022, an estimated 20 million new cancer cases were diagnosed globally in 2022, including nonmelanoma skin cancers (NMSC). The top six cancer types in both sexes together accounted for more than half of the total cancer burden. Lung cancer remained the most commonly diagnosed cancer worldwide, with 2.48 million new cases (12.4% of the total), followed by cancers of the female breast (11.6%), colorectum (9.6%), prostate (7.3%), stomach (4.8%), and liver (4.3%) [2]. Liver cancer has consistently ranked as the sixth most commonly diagnosed malignancy worldwide in the GLOBOCAN datasets for 2012, 2018, 2020, and 2022 [1, 2, 3, 4]. In 2022, there were approximately 865,000 newly diagnosed liver cancer cases (Figure 1A). Over the past decade, the crude number of new cases fluctuated: 783,000 in 2012, 841,000 in 2018, 906,000 in 2020, and 865,000 in 2022. Despite these variations, the proportion of liver cancer among all newly diagnosed cancers has shown a modest declining trend, with 5.6% in 2012, 4.7% in 2018, 4.7% in 2020, and 4.3% in 2022 (Figure 1B). The global age‐standardized incidence rate (ASIR) of liver cancer in 2022 was 8.6 per 100,000, ranking it eighth among all cancer types (Figure 1C). According to GBD data, the ASIR for liver cancer has shown an overall declining trend from 1990 to 2020, mirroring trends in crude incidence. However, two modest peaks were observed in 2000 and 2014, with ASIRs of 6.7 and 6.5 per 100,000, respectively (Figure 1D). Sex‐based disparities in liver cancer incidence remain pronounced. GLOBOCAN 2022 reported a substantially higher ASIR in males (12.7 per 100,000) compared to females (4.8 per 100,000) (Figure 1E). These differences are partially attributable to behavioral factors such as higher rates of alcohol use, smoking, and HBV infection in males, but may also be linked to sex hormone regulation and preclinical studies showing greater tumor susceptibility in male mice [21, 22, 23]. Liver cancer incidence also exhibits a marked age dependency. The ASIR peaks in individuals aged 75–84 years (59.8 per 100,000), followed by 60–74 years (43.2 per 100,000), and rates in all other age groups were below 20 per 100,000 (Figure 1F). This trend aligns with the biological hallmarks of aging, which include genomic instability, epigenetic modifications, chronic inflammation, and gut dysbiosis, all of which collectively increase cancer susceptibility [24, 25].

According to *The Lancet Commission*, if the current ASIR remains unchanged, global liver cancer cases are projected to rise to 1.52 million annually by 2050. To counter this trajectory, a minimum annual reduction in ASIR of 2% is required. Achieving a 2.0%–5.0% annual decline could prevent up to 17.3 million new cases and save an estimated 15.1 million lives by 2050 [6].

### 3.2 New Cases and Incidence Trends in China

**Figure 2:**
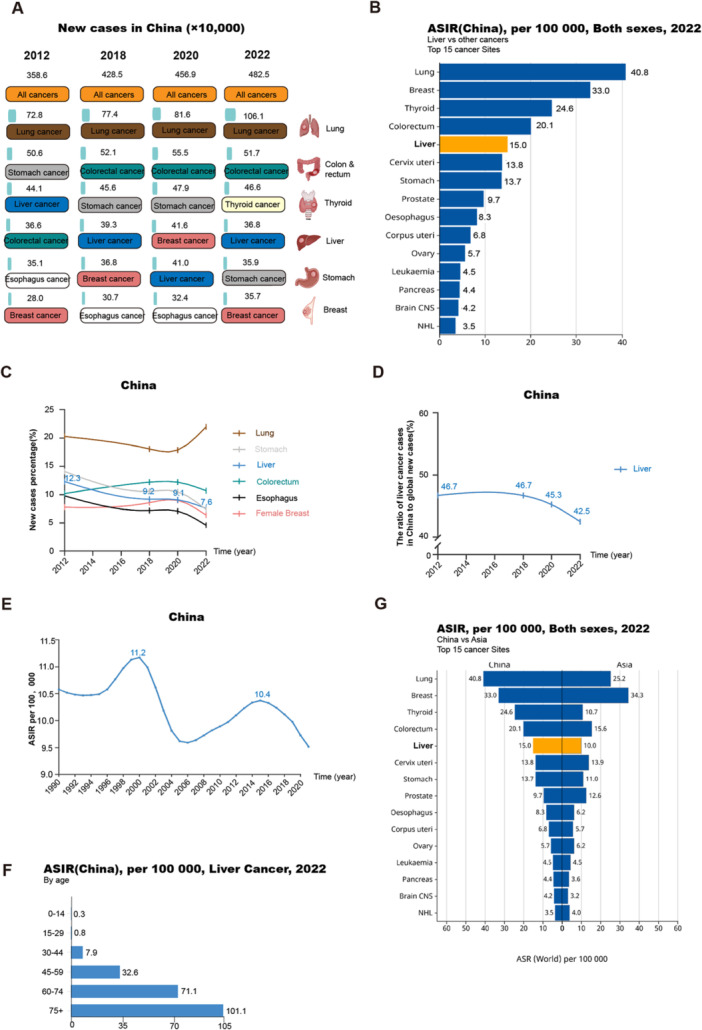
Temporal trends in liver cancer incidence in China. (A) Newly diagnosed liver cancer cases in 2012, 2018, 2020, and 2022, based on GLOBOCAN data. (B) The 15 most common cancers ranked by ASIR with liver cancer highlighted based on GLOBOCAN 2022 data. (C) Proportion of liver cancer among all new cancer cases in China in 2012, 2018, 2020, and 2022 (GLOBOCAN data); no data were available for 2014 and 2016. (D) China′s contribution to global liver cancer incidence across the same years (GLOBOCAN data); no data were available for 2014 and 2016. (E) Temporal trends in liver cancer ASIR in China from 1990 to 2021, based on GBD data. (F) Age‐specific ASIR distribution of liver cancer in China (GLOBOCAN 2022 data). (G) Comparison of ASIR rankings for the top 15 cancers between China and the broader Asian region, with liver cancer highlighted (GLOBOCAN 2022 data). (B) and (G) share the same map source: World Health Organization (https://gco.iarc.who.int/today/). ASIR, age‐standardized incidence rate; CNS, central nervous system; GBD, Global Burden of Disease; NHL, non‐Hodgkin lymphoma.

According to GLOBOCAN 2022 data, China recorded an estimated 4.8 million newly diagnosed cancer cases in 2022, including NMSC (Figure 2A). The top six cancer types in both sexes accounted for approximately 64.8% of all newly diagnosed cancers. Lung cancer remained the most frequently diagnosed cancer, with 1.06 million new cases, representing 22.0% of all cases, followed by colorectal cancer (10.7%), thyroid cancer (9.7%), liver cancer (7.6%), stomach cancer (7.4%), and female breast cancer (7.4%). Liver cancer was the fourth most commonly diagnosed malignancy in China in 2022, with an estimated 368,000 new cases [14]. The number of newly diagnosed liver cancer cases has shown a fluctuating downward trend over the past decade, from 441,000 in 2012 to 393,000 in 2018, 410,000 in 2020, and 368,000 in 2022 (Figure 2A). The ASIR of liver cancer in China is approximately 15.0 per 100,000, ranking fifth among all tumor types (Figure 2B), and remains substantially significantly higher than the global ASIR of 8.6 per 100,000. While the global proportion of liver cancer among newly diagnosed cancers has declined only modestly, China has experienced a more pronounced and consistent downward trend. The proportion of new cancer cases attributed to liver cancer fell from 12.3% in 2012 to 9.2% in 2018, 9.1% in 2020, and 7.6% in 2022 (Figure 2C). Within the global burden of liver cancer, China's share of new cases remained high, but showed a gradual downward trend over the period, from 46.7% in 2012 to 46.7% in 2018, 45.3% in 2020, and 42.5% in 2022. These temporal changes in proportion mirror the declining national incidence rate, underscoring that liver cancer continues to pose a major public health challenge despite recent progress (Figure 2D). Although China′s proportion of new liver cancer cases relative to global totals has decreased, it still accounts for over 40% of all new liver cancer cases worldwide (Figure 2D). According to data from the GBD database, the ASIR of liver cancer in China demonstrated a fluctuating but overall downward trend from 1990 to 2021. Two small peaks were observed during this period: in 2000 and 2015, with ASIRs of 11.2 and 10.4 per 100,000, respectively (Figure 2E). China also exhibits a clear sex‐based disparity in liver cancer incidence. As reported in GLOBOCAN 2022, the ASIR among males is substantially higher than among females, at 22.7 per 100,000 and 7.4 per 100,000, respectively. The age distribution of liver cancer in China also parallels global trends. The ASIR increases markedly with age, reaching 71.1 per 100,000 in individuals aged 60–74 years, and peaking at 101.1 per 100,000 in those aged 75 years and older (Figure 2F). Asia remains a region with a high burden of liver cancer [26]. Notably, China′s ASIR significantly exceeds the average ASIR for the Asian region (Figure 2G), highlighting the urgency of addressing the liver cancer epidemic through intensified prevention, early detection, and control measures [27].

### 3.3 Geographic Patterns of Liver Cancer Epidemiology

**Figure 3:**
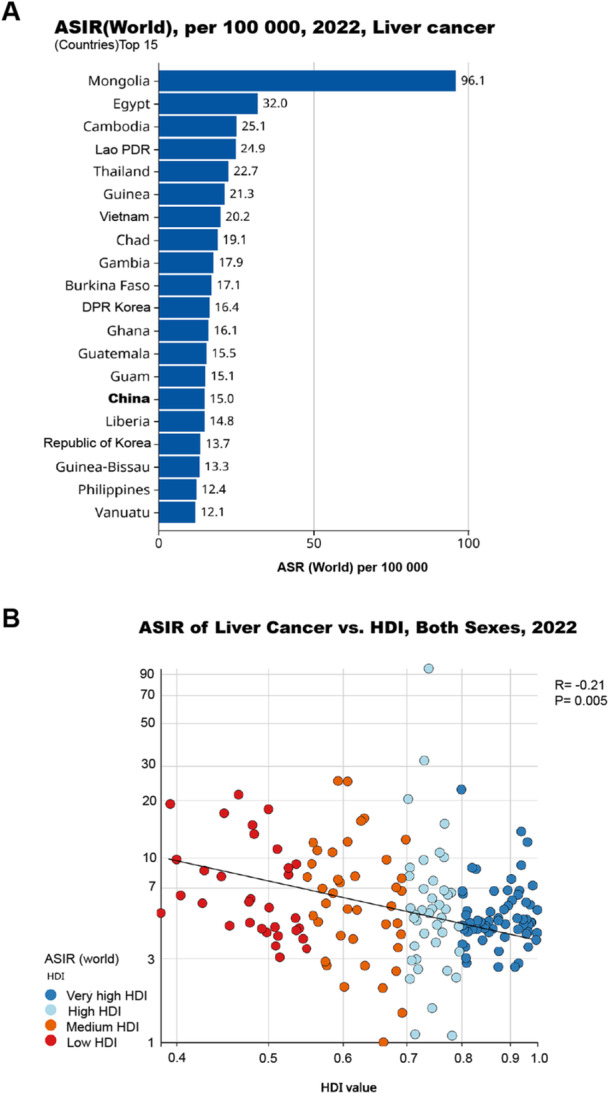
Global geographical patterns and socioeconomic correlates of liver cancer incidence. (A) Global distribution of liver cancer incidence represented by ASIR across countries, based on GLOBOCAN 2022 estimates. (B) Inverse correlation between national Human Development Index (HDI) scores and liver cancer ASIR, illustrating the disproportionate burden of liver cancer in low‐HDI countries, based on GLOBOCAN 2022 data. The two figures share the same resource: World Health Organization (https://gco.iarc.who.int/today/). ASIR, age‐standardized incidence rate; DPR Korea, Democratic People's Republic of Korea; Lao PDR, Lao People's Democratic Republic.

Globally, liver cancer incidence exhibits marked geographic variation. According to GLOBOCAN 2022 data, the top 15 highest ASIRs were reported in Mongolia (96.1 per 10,000), followed by countries such as Egypt (32.0 per 100,000), Cambodia (25.1 per 100,000), Lao People′s Dem. (24.9 per 100,000), and Thailand (22.7 per 10,000) (Figure 3A). Notably, the ASIR in China also remains at a relatively high level (15.0 per 10,000) (Figure 3A). In contrast, regions such as North America and Western Europe report significantly lower ASIRs, at approximately 6.7 and 5.5 per 100,000 people, respectively [27]. Additionally, the ASIR of liver cancer is inversely associated with a region′s level of socioeconomic development, often indexed by the Human Development Index (HDI). Regions with relatively higher HDI scores, such as Europe, Oceania, and North America, tend to have lower liver cancer incidence rates (Spearman′s correlation: *r* = −0.21, *p* = 0.005), whereas regions with lower HDI scores, including many parts of Africa, Asia, and Latin America, exhibit substantially higher rates (Figure 3B). This disparity may be attributed to differences in better healthcare infrastructure, vaccination coverage, screening program accessibility, and the prevalence of major risk factors such as HBV infection. In China, liver cancer demonstrates distinct regional patterns. The guidelines issued by the China Anti‐Cancer Association (CACA) delineate geographic disparities in incidence, identifying the southeastern coastal provinces, particularly Jiangsu, Zhejiang, Guangdong, and Guangxi, as high‐incidence areas. Within these regions, estuarine and island zones show especially elevated rates. Contributing factors include high population density, which facilitates HBV transmission, and humid climatic conditions that promote the growth of mold and aflatoxin contamination in food. Furthermore, liver cancer incidence rates tend to be higher in rural areas compared to urban centers, with a more pronounced rural–urban disparity observed in eastern China [28]. This discrepancy may stem from differences in lifestyle, occupational exposures, socioeconomic status, and access to preventive services such as HBV vaccination and cancer screening [29]. Regional dietary practices, environmental toxins, and health literacy may also contribute to these patterns.

## 4 Mortality Trends of Liver Cancer

### 4.1 Death Cases and Mortality Trends Worldwide

**Figure 4:**
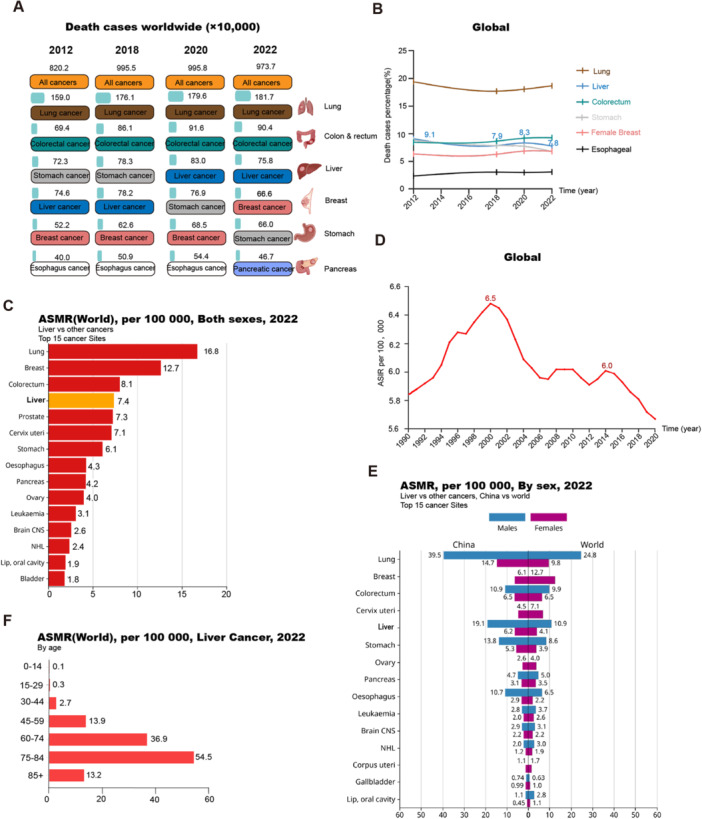
Global temporal trends in liver cancer mortality. (A) Annual number of liver cancer deaths worldwide in 2012, 2018, 2020, and 2022, based on GLOBOCAN estimates. (B) Proportion of global liver cancer deaths occurring in China across the same years based on GLOBOCAN estimates; no data were available for 2014 and 2016. (C) Ranking of the top 15 cancers by ASMR globally in 2022, with liver cancer highlighted for comparison (GLOBOCAN 2022 data). (D) Temporal trends in global liver cancer ASMR from 1990 to 2021, derived from GBD data. (E) Comparison of ASMR for liver cancer between China and the global population, stratified by sex (GLOBOCAN 2022 data). (F) Age‐specific ASMR distribution for liver cancer worldwide, based on GLOBOCAN 2022 data. (C) and (E) share the same map source: World Health Organization (https://gco.iarc.who.int/today/). ASMR, age‐standardized mortality rate; CNS, central nervous system; GBD, Global Burden of Disease; NHL, non‐Hodgkin lymphoma.

Liver cancer mortality is closely linked to its incidence, but it can be significantly reduced through early detection and timely, effective treatment. Therefore, while regions with high incidence generally bear a high mortality burden, the quality of diagnostic and therapeutic interventions plays a critical role in moderating overall death rates. According to GLOBOCAN 2022 estimates, liver cancer ranked as the third leading cause of cancer‐related deaths globally, following lung and colorectal cancers. In 2022, approximately 758,000 deaths were attributed to liver cancer (Figure 4A), imposing a considerable disease and economic burden on populations worldwide [2]. An analysis of temporal trends reveals a gradual decline in liver cancer′s share of total global cancer deaths, decreasing from 9.1% in 2012 to 7.8% in 2022, which mirrors the downward trend in its contribution to overall cancer incidence (Figure 4B). After adjustment for age, the age‐standardized mortality rate (ASMR) for liver cancer is approximately 7.4 per 100,000, ranking fourth globally after lung, breast, and colorectum cancers (Figure 4C). Historical ASMR data indicate that liver cancer mortality peaked around 2000, with an ASMR of 6.5 per 100,000. This was followed by a fluctuating decline, including a modest rebound to 6.0 per 100,000 in 2014, and a more consistent downward trend in subsequent years (Figure 4D). Significant gender disparities in liver cancer mortality are also observed globally. The ASMR for males is 10.9 per 100,000, which is more than twice that for females, at 4.1 per 100,000 (Figure 4E). This difference may be attributed to variations in exposure to major risk factors such as HBV infection, alcohol consumption, and metabolic diseases, as well as biological influences, including sex hormones and immune responses. Age‐specific mortality data further emphasize the disproportionate impact of liver cancer on older populations. The highest ASMR is found in individuals aged 75–84 years (54.5 per 100,000), followed by the 60–74 age group (36.9 per 100,000), while a notable decline is observed in those aged 85 years and older, with an ASMR of 13.2 per 100,000 (Figure 4F). This age‐related pattern aligns with established oncogenic mechanisms associated with aging, such as genomic instability, epigenetic drift, and chronic inflammation.

**Figure 5:**
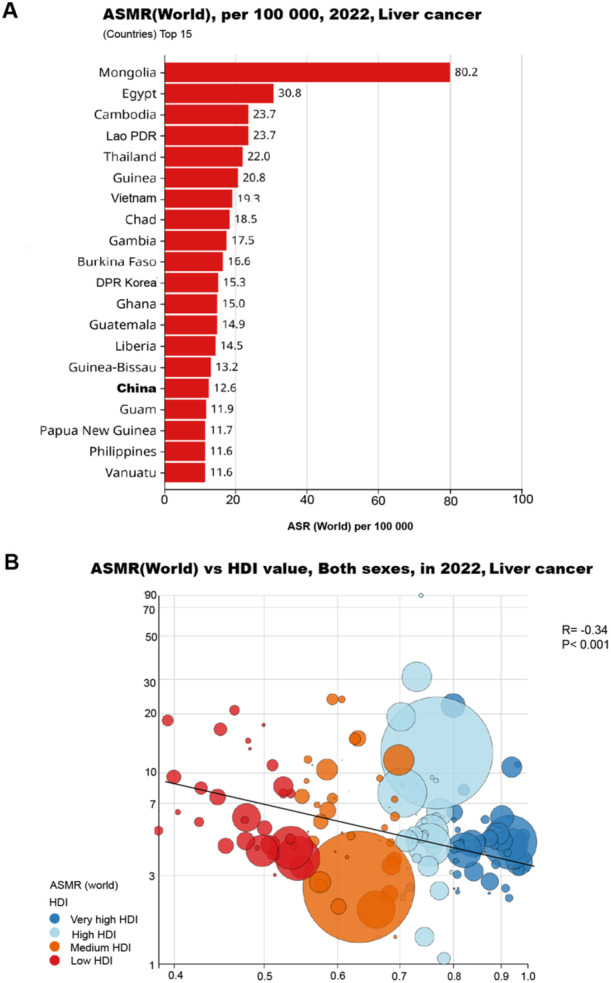
Global geographical variation in liver cancer mortality and its association with socioeconomic development. (A) ASMR for liver cancer across countries, based on GLOBOCAN 2022 estimates. (B) Global correlation between national ASMR for liver cancer and the HDI based on GLOBOCAN 2022 data, illustrating the inverse association between mortality and socioeconomic development. Population size was incorporated, with circle size scaled proportionally to the population. The two figures share the same resource: World Health Organization (https://gco.iarc.who.int/today/). ASMR, age‐standardized mortality rate; HDI, Human Development Index; Lao PDR, Lao People's Democratic Republic.

**Figure 6:**
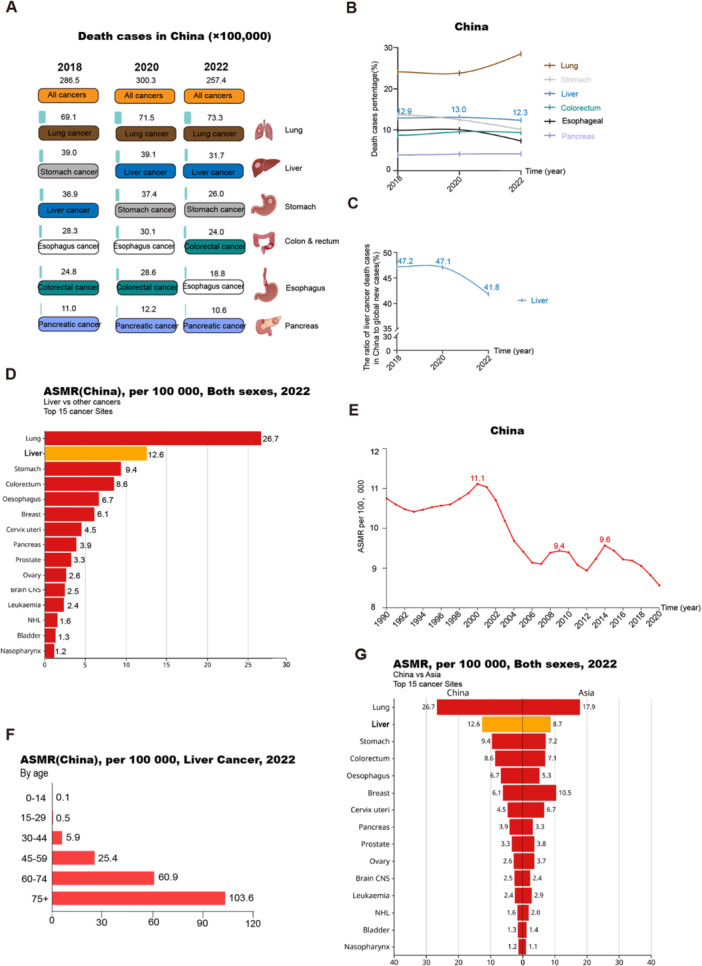
Mortality burden and temporal trends of liver cancer in China. (A) Number of liver cancer deaths in China in 2018, 2020, and 2022 based on GLOBOCAN estimates. (B) Proportion of liver cancer‐related deaths among all cancer deaths in China in 2018, 2020, and 2022 based on GLOBOCAN estimates. (C) China′s contribution to global liver cancer deaths in 2018, 2020, and 2022 based on GLOBOCAN estimates. (D) Ranking of the top 15 cancers by ASMR in China for both sexes, based on GLOBOCAN 2022 data, highlighting liver cancer. (E) Temporal trends in liver cancer ASMR in China from 1990 to 2021 using data from the GBD study. (F) Age‐specific distribution of liver cancer ASMR in China based on GLOBOCAN 2022 data. (G) Comparison of the top 15 cancers by ASMR in China versus the broader Asian region, with liver cancer highlighted based on GLOBOCAN 2022 data. (D) and (G) share the same map source: World Health Organization (https://gco.iarc.who.int/today/). ASMR, age‐standardized mortality rate; CNS, central nervous system; GBD, Global Burden of Disease; NHL, non‐Hodgkin lymphoma.

Similar to the ASIR, the ASMR exhibits a negative correlation with the HDI. Regions with higher HDI values, such as Europe and North America, generally have lower ASMRs. In contrast, areas with lower HDI values, including East Asia and Africa, particularly Mongolia, Egypt, Cambodia, Lao People's Democratic Republic, and Thailand report ASMRs exceeding 8.0 per 100,000. Meanwhile, high‐HDI regions such as North America and Western Europe often show ASMRs below 5.1 per 100,000, with some Western European countries specifically ranging between 1.1 and 3.3 per 100,000 (Figure 5, 6) (Spearman′s correlation between HDI and ASMR: *r* = −0.34, *p* < 0.001). Countries with higher HDI and Gross Domestic Product (GDP) per capita exhibit lower liver cancer ASMR, attributable to socioeconomic factors like lower poverty and higher education levels, which similarly mediate the HDI–ASIR association [30]. Since 1990, a decline in HBV and HCV seroprevalence has contributed to decreasing liver cancer mortality in East Asian countries like Japan and China. Conversely, countries with previously low liver cancer risk, particularly in Europe and North America, have experienced rising mortality rates. This increase is attributed to the growing burden of metabolic risk factors, including diabetes, obesity, and non‐alcoholic fatty liver disease (NAFLD) [31, 32]. The global burden of liver cancer continues to pose a major public health challenge. Without proactive prevention strategies, the number of liver cancer deaths is projected to increase from 760,000 in 2022 to 1.37 million by 2050 [6].

### 4.2 Death Cases and Mortality Trends in China

China continues to bear a disproportionately high burden of liver cancer, which remains the second leading cause of cancer‐related mortality in the country. In 2022, there were approximately 317,000 crude liver cancer deaths in China, accounting for 36.8% of the global total (Figure 6A). The proportion of liver cancer deaths among all cancer‐related deaths in China declined fluctuatingly from 12.9% in 2018 to 12.3% in 2022, yet it still ranked second only to lung cancer in 2022 (Figure 6B). Although the proportion of liver cancer deaths in China relative to the global total has shown a downward trend, it continues to remain alarmingly high and consistently exceeds 40.0%, despite national progress in hepatitis B vaccination and medical advancements [20, 33, 34, 35]. This persistent burden reflects both the historical prevalence of HBV and the emerging influence of metabolic risk factors (Figure 6C). After age standardization, the ASMR for liver cancer in China was 12.6 per 100,000 in 2022, ranking second among all cancers, only followed lung cancer (26.7 per 100,000) (Figure 6D). The national ASMR peaked in 2000 at 11.1 per 100,000 and subsequently exhibited a fluctuating decline, with minor rebounds in 2009 and 2014 when ASMRs reached 9.4 and 9.6 per 100,000, respectively (Figure 6E). The ASMR of liver cancer in China rose sharply with age (Figure 6F). As reported in earlier studies, HCC is the fifth most commonly diagnosed and second most fatal cancer in Asia [36]. According to GLOBOCAN 2022 data, China′s liver cancer ASMR of 12.6 per 100,000 significantly exceeds the Asian regional average of 8.7 per 100,000, highlighting the need for intensified intervention (Figure 6G). In response to this public health crisis, hepatologists in China have made substantial efforts to improve prevention, early diagnosis, and therapeutic strategies [6, 28, 37, 38], ieading to improved prognosis of liver cancer in China. Data from a major clinical center, Zhongshan Hospital of Fudan University, revealed that from 2011 to 2020, patients undergoing radical liver cancer resection achieved 5‐ and 10‐year survival rates of 70.7% and 57.8%, respectively. Importantly, nearly 3500 patients survived more than 10 years post‐surgery, placing these outcomes among the highest globally. In contrast, during earlier periods such as 1958–1980, the 5‐ and 10‐year survival rates were only 10.1% and 7.3% respectively. These improvements highlight the critical impact of advances in surgical techniques, perioperative management, and long‐term follow‐up care [6].

The ASMR of liver cancer in China exhibits significant inter‐provincial geographical disparities. High‐burden provinces are primarily concentrated in the southeastern coastal regions and northeastern China, with rates generally higher among males than females. However, the overall distribution patterns across regions are broadly similar between males and females. For details, please refer to Table 1 [39]. The geographical areas with high age‐standardized years of life lost rates (ASYLRs) (per 100,000) for liver cancer in China show substantial overlap with those regions characterized by high ASMRs. For details, please refer to Table 2 [39].

**Table 1:** ASMR of HCC across different provinces in china, by sex.

Men		Women	
Province	ASMR (per 100,000)	Province	ASMR (per 100,000)
Guangxi	50.2	Heilongjiang	16.6
Heilongjiang	43.4	Jilin	13.4
Guangdong	41.9	Henan	13.2
Hainan	39.3	Ningxia	12.9
Fujian	38.4	Chongqing	12.6
Chongqing	34.9	Neimenggu	12.0
Jilin	34.2	Guangxi	12.0
Jiangxi	33.1	Sichuan	11.7
Henan	32.9	Liaoning	11.7
Sichuan	31.6	Fujian	11.6
Zhejiang	31.5	Hubei	11.4
Liaoning	31.2	Anhui	11.0
Hubei	30.6	Shandong	11.0
Shandong	29.7	Jiangsu	11.0
Anhui	28.9	Zhejiang	10.8
Jiangsu	28.4	Jiangxi	10.8
Neimenggu	28.4	Shaanxi	10.8
Hunan	27.4	Guangdong	10.7
Ningxia	24.3	Qinghai	10.6
Yunnan	22.9	Gansu	10.5
Qinghai	22.7	Shanxi	10.5
Shaanxi	21.1	Hunan	10.2
Hebei	20.8	Hainan	8.9
Shanghai	20.5	Hebei	8.7
Gansu	19.8	Yunnan	8.0
Shanxi	19.7	Shanghai	7.8
Guizhou	19.6	Xinjiang	7.7
Xizang	17.1	Guizhou	7.6
Tianjin	15.3	Xizang	7.0
Beijing	15.2	Beijing	6.8
Xinjiang	13.2	Tianjin	6.6

*Note:* The data in this table are derived from the Chinese National Death Surveillance System (CNDSS) for the period 2013–2020 [39]. In this table, “provinces” includes autonomous regions and municipalities directly under the Central Government.

Abbreviations: HCC, hepatocellular carcinoma; ASMR, age‐standardized mortality rate.

**Table 2:** ASYLL (per 100,000) of liver cancer in China, 2013–2017.

ASYLL (per 100,000)	Year				
	2013	2014	2015	2016	2017
North	230.2	266.8	258.2	260.2	254.5
Northeast	494.9	532.8	522.0	519.0	486.8
Northwest	224.1	263.3	292.7	285.7	294.6
Central	384.0	469.5	471.0	441.8	443.2
East	453.1	461.5	466.0	433.6	426.2
South	540.9	647.4	676.2	666.2	658.7
Southwest	439.8	484.0	474.1	451.8	438.3

*Note:* The data in this table are derived from the Chinese National Death Surveillance System (CNDSS) for the period 2013–2017 [39].

Abbreviation: ASYLL, age‐standardized years of life lost rate.

## 5 Factors Influencing Incidence and Mortality Trends of Liver Cancer

### 5.1 Chronic Viral Hepatitis

**Figure 7:**
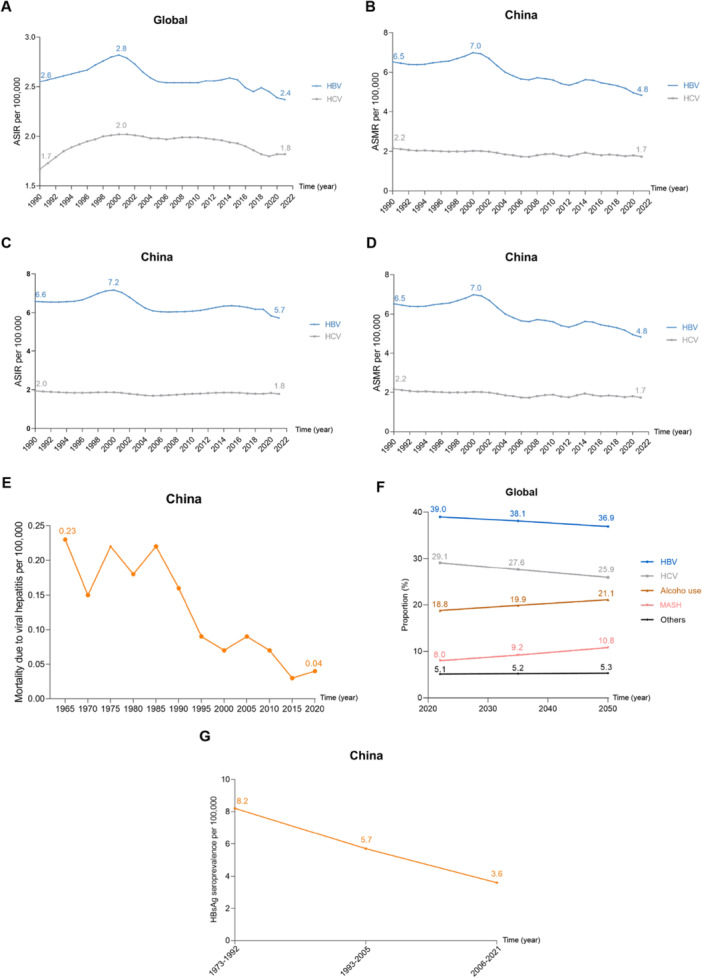
Etiology‐specific incidence and mortality patterns of liver cancer in China and worldwide. (A) Global ASIR of HBV‐ and HCV‐related liver cancer from 1990 to 2021 (GBD data). (B) Global ASMR of HBV‐ and HCV‐related liver cancer during the same period (GBD data). (C) ASIR of HBV‐ and HCV‐related liver cancer in China from 1990 to 2021 (GBD data). (D) ASMR of HBV‐ and HCV‐related liver cancer in China from 1990 to 2021 (GBD data). (E) Mortality rates associated with viral hepatitis in China from 1965 to 2021, derived from the China Health Statistics Yearbook 2022. (F) Projected shifts in liver cancer etiologic composition for the years 2022, 2035, and 2050 [6]. (G) Temporal trends in hepatitis B surface antigen (HBsAg) positivity prevalence in China from 1973 to 2021, based on the China Health Statistics Yearbook 2022. ASIR, age‐standardized incidence rate; ASMR, age‐standardized mortality rate; GBD, Global Burden of Disease; HBV, hepatitis B virus; HCV, hepatitis C virus; MASH, metabolic dysfunction‐associated steatohepatitis.

HCC is predominantly associated with chronic infection by HBV or HCV, as well as alcohol‐related liver disease and MASLD. Other established cofactors include aflatoxin exposure, obesity, and diabetes. Among these, HBV and HCV remain the leading causes of HCC globally. In 2021, there were 206,366 newly diagnosed cases of HBV‐related HCC (39.4% of the total), and 154,062 cases were attributed to HCV‐related HCC (30.3%) [40]. Between 2010 and 2021, the global number of new HBV‐related HCC cases increased by 20.8%, while HCV‐related cases rose by 24.7% [40]. This increase is largely attributed to population aging. However, after age standardization, the ASIR of HBV‐related HCC showed substantial declines from 1990 to 2021, with annual percentage decline of 0.3% (Table 3) [40]. The annual percentage change (APC) of HCV‐related HCC is not significant from 1990 to 2021 (Table 3). Among all etiological types, HBV‐related HCC demonstrated the most significant ASIR reduction, declining from 2.8 per 100,000 in 2000 to 2.4 per 100,000 in 2021 (Figure 7A). Similarly, HCV‐related HCC decreased from 2.0 per 100,000 in 2001 to 1.8 per 100,000 in 2021 (Figure 7A). In terms of crude mortality, HBV‐related HCC caused 181,194 deaths globally in 2021 (37.3%), while HCV‐related HCC accounted for 146,522 deaths (31.0%), jointly comprising 68.3% of global HCC‐related mortality [40]. Due to aging populations, the global absolute number of deaths from HBV‐related HCC increased by 17.0%, and HCV‐related death cases increased by 27.0% between 2010 and 2021 [40]. After age standardization, the ASMR for HBV‐related HCC declined by 0.65% annually, and by 0.08% annually for HCV‐related HCC (Table 3). From 1990 to 2021, the ASMR for HBV‐related HCC decreased with fluctuations from 2.5 to 2.1 per 100,000, peaking at 2.7 per 100,000 in 2000. The ASMR for HCV‐related HCC also declined, from 2.0 to 1.7 per 100,000 between the year 2000 and 2021 (Figure 7B). In China, the ASIR for HBV‐related HCC declined with fluctuations from 6.6 to 5.7 per 100,000 between 1990 and 2021, with peaks at 7.2 per 100,000 in 2000 and 6.4 per 100,000 in 2014 and the APC was −0.41 from 1990 to 2021 (Table 4). The ASIR for HCV‐related HCC fluctuated between 2.0 and 1.8 per 100,000 without distinct peaks from 1990 to 2021 (Figure 7C) and the APC was −0.15 in this period (Table 4). The ASMR for HBV‐related HCC in China varied from 6.5 to 4.8 per 100,000, with a peak of 7.0 per 100,000 in 2000. The ASMR for HCV‐related HCC declined from 2.2 to 1.7 per 100,000, without notable increases during the same timeframe (Figure 7D). The APCs for HBV‐related HCC and HCV‐related HCC were −0.97 and −0.58, respectively, from 1990 to 2021 (Table 4). Data from the *China Health Statistics Yearbook* further indicate a consistent decrease in both the ASMR of HBV‐related HCC and deaths from other severe HBV‐related liver diseases, such as fulminant viral hepatitis [41] and virus‐associated acute‐on‐chronic liver failure [42] (Figure 7E).

**Table 3:** Trends in etiology‐specific ASIRs and ASMRs worldwide from 1990 to 2021.

Etiology	APC	CI	*p* value
ASIRs			
HBV	−0.30	−0.44, −0.16	< 0.001
HCV	0.03	−0.18, 0.24	0.780
Alcohol use	0.56	0.47, 0.65	< 0.001
MASH	0.96	0.86, 1.07	< 0.001
Others	−0.40	−0.51, −0.28	< 0.001
ASMRs			
HBV	−0.65	−0.80, −0.50	< 0.001
HCV	−0.08	−0.27, −0.11	0.380
Alcohol use	0.31	−0.23, 0.39	< 0.001
MASH	0.79	0.69, 0.89	< 0.001
Others	−0.65	−0.76, −0.54	< 0.001

*Note:* The data in this table (e.g., APC values) are derived from the GBD 1990–2021 dataset and were analyzed using Joinpoint regression.

Abbreviations: ASIR, age‐standardized incidence rate; ASMR, age‐standardized mortality rate; APC, annual percentage change; CI, confidence interval; GBD, Global Burden of Disease; HBV, hepatitis B virus; HCV, hepatitis C virus; MASH, metabolic dysfunction‐associated steatohepatitis.

**Table 4:** Trends in etiology‐specific ASIRs and ASMRs in China from 1990‐2021.

Etiology	APC	CI	*p* value
ASIRs			
HBV	−0.41	−0.62, −0.20	< 0.001
HCV	−0.15	−0.27, −0.02	0.020
Alcohol use	0.64	0.43, 0.85	< 0.001
MASH	0.71	0.49, 0.93	< 0.001
Others	−1.22	−1.48, −0.95	< 0.001
ASMRs			
HBV	−0.97	−1.19, −0.75	< 0.001
HCV	−0.58	−0.72, −0.43	< 0.001
Alcohol use	0.16	−0.03, 0.35	0.110
MASH	0.79	0.69, 0.89	< 0.001
Others	−0.03	−0.21, −0.15	0.690

*Note:* The data in this table (e.g., APC values) are derived from the GBD 1990–2021 dataset and were analyzed using Joinpoint regression.

Abbreviations: ASIR, age‐standardized incidence rate; ASMR, age‐standardized mortality rate; APC, annual percentage change; CI, confidence interval; GBD, Global Burden of Disease; HBV, hepatitis B virus; HCV, hepatitis C virus; MASH, metabolic dysfunction‐associated steatohepatitis.

Based on data from the GBD Study (1990–2021), a Chinese research team projected the etiological distribution of HCC for 2022, 2035, and 2050. Their model predicts that by 2050, the proportions of HCC attributable to HBV and HCV will decline from 39.0% to 36.9%, and from 29.1% to 25.9%, respectively (Figure 7F). The continued decline in the incidence and mortality of viral hepatitis‐related HCC can be attributed to several public health interventions, including the implementation of universal HBV vaccination programs in the 1980s, the availability of antiviral therapies, the use of HCV direct‐acting antivirals (DAAs), and environmental health policies aimed at reducing exposure to aflatoxins and other carcinogens [43, 44, 45]. A recent meta‐analysis estimated the seroprevalence of HBV and HCV in patients with HCC to be 75.1% (95% confidence interval (CI): 73.1%–77.0%) and 11.82% (95% CI: 9.8%–14.0%), respectively. After adjustments, the estimated HBV seroprevalence was 64.1%, notably higher than the global average of 56.0% [18]. China′s HBV prevention programs have yielded substantial success. Between 1973 and 2021, the seroprevalence of HBV in the population decreased from 8.2 to 3.6 per 100,000, while hepatitis‐related deaths declined from 0.23 to 0.04 per 100,000 (Figure 7G).

### 5.2 Metabolic Dysfunction‐Associated Steatotic Liver Disease (MASLD)

MASLD, formerly known as NAFLD, encompasses a spectrum of liver disorders associated with metabolic dysfunction, with hepatic steatosis as the central pathological feature [46]. MASLD is defined by the presence of metabolic dysfunction rather than exclusion of other liver diseases, such as alcohol‐induced liver disease or viral hepatitis, and it can coexist with other etiologies [47]. Diagnosed is typically based on ultrasonographic evidence of hepatic steatosis, following the exclusion of significant alcohol consumption, steatogenic drug use, and other chronic liver disease [48]. The hallmark of MASLD is the pathological accumulation of lipids, predominantly triglycerides, in hepatocytes. This lipid overload stimulates increased fatty acid oxidation and oxidative stress, resulting in lipid peroxidation, mitochondrial dysfunction, and the release of pro‐inflammatory mediators. These pathophysiological events drive the progression from simple steatosis to steatohepatitis and ultimately fibrosis [49]. The prevailing pathogenesis model is the “multiple parallel hits” hypothesis, which implicates systemic metabolic dysregulation as a key contributor to disease progression [50]. Accumulating evidence demonstrates that steatohepatitis, particularly when accompanied by hepatic fibrosis, markedly increases the risk of adverse outcomes, including cirrhosis and HCC [51]. A subset of MASLD known as MASH represents a more advanced and inflammatory stage of the disease [52]. In this stage, hepatic steatosis is accompanied by hepatocellular injury and inflammation. It is estimated that 23% of patients with MASLD progress to MASH within 3 years [52]. Moreover, approximately 2% of MASH patients develop MASH‐related HCC annually [53]. Studies have shown that the weak carcinogenicity of MASLD can be significantly activated in the presence of viral hepatitis. Using populations without MASLD or viral hepatitis as the reference group, after 15 years of follow‐up, the cumulative incidence of HCC was 0.3% in the reference group, 4.4% in patients with viral hepatitis alone, 1.5% in patients with MASLD alone, and 5.5% in patients with both MASLD and viral hepatitis [54].

**Figure 8:**
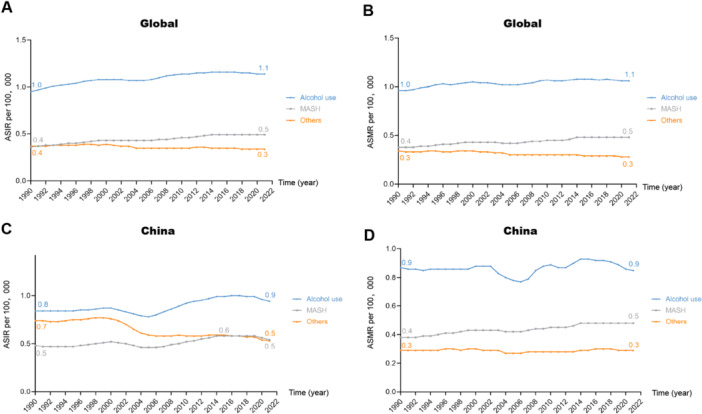
ASIR and ASMR of liver cancer attributable to alcohol use and MASH in China and globally, from 1990 to 2021, based on the GBD database. (A) Global ASIR of liver cancer attributed to alcohol use and MASH. (B) Global ASMR of liver cancer attributed to alcohol use and MASH. (C) ASIR of alcohol‐related and MASH‐related liver cancer in China. (D) ASMR of alcohol‐ related and MASH‐related liver cancer in China. ASIR, age‐standardized incidence rate; ASMR, age‐standardized mortality rate; GBD, Global Burden of Disease; MASH, metabolic dysfunction‐associated steatohepatitis.

Globally, the prevalence of MASLD exceeds 38.8% [48]. Between 2010 and 2021, the ASIR of MASLD‐related liver cancer increased steadily, with an APC of +0.7% (95% uncertainty interval [UI]: 0.6–0.7). A similar upward trend was observed for the ASMR, which rose with an APC of +0.6% (95% UI: 0.4–0.9). These increases were especially in the Americas, Southeast Asia, and the Eastern Mediterranean [40]. Based on data from the GBD Study (1990–2021), the global ASIR of MASH‐related HCC increased from 0.4 to 0.5 per 100,000, while the ASMR rose in parallel from 0.4 to 0.5 per 100,000 (Figure 8A,B). In China, the ASIR of MASH‐related liver cancer remained relatively stable over the past two decades; however, the ASMR showed an increase from 0.4 to 0.5 per 100,000 (Figure 8C,D). These trends highlight the emerging importance of MASLD and its inflammatory subtype MASH as etiological drivers of liver cancer. Consequently, MASLD‐related HCC warrants increased attention in public health policy, clinical surveillance, and targeted prevention strategies.

### 5.3 Aflatoxins Exposure in Environment and Diet

Aflatoxins are mycotoxins produced as secondary metabolites by *Aspergillus* species, particularly *Aspergillus flavus*. Among the various aflatoxin subtypes, Aflatoxin B1 (AFB1) is the most prevalent and most potent [55]. AFB1 exerts multiple toxic effects in mammals and poultry, including carcinogenicity [56], DNA damage [57], immunotoxicity [58], and hepatotoxicity [59]. Acute exposure to high doses of aflatoxins can result in aflatoxicosis, whereas long‐term ingestion of low doses is strongly linked to the development of HCC [60]. Chronic exposure may also occur through the consumption of eggs, dairy products, and animal organs, particularly the liver, derived from livestock fed aflatoxin‐contaminated feed [61, 62]. AFB1 exposure is estimated to account for 4.6% to 28.2% of global HCC cases. Moreover, co‐exposure to HBV dramatically amplifies carcinogenic risk; individuals with chronic HBV infection who are exposed to AFB1 have a 30‐fold higher risk of developing HCC compared with unexposed individuals [63, 64, 65]. This synergistic effect highlights the importance of addressing both environmental toxin exposure and viral hepatitis in regions with high HCC incidence. A broad range of agricultural commodities, including cereals, nuts, and dairy products, are vulnerable to aflatoxin contamination at multiple stages, ranging from pre‐harvest to long‐term storage [66, 67]. Contamination risk increases under environmental conditions that favor Aspergillus growth, such as sustained high temperatures, elevated humidity, and the absence of prolonged cold periods [68]. As a result, countries in tropical and subtropical regions, particularly in Sub‐Saharan Africa and Southeast Asia, experience disproportionately high levels of aflatoxin exposure [66, 67]. In China, aflatoxin exposure is more prevalent in southeastern regions, including Guangdong and Fujian provinces, where climatic conditions facilitate fungal growth. These regions report higher rates of aflatoxin contamination in food supplies, which correspond to increased incidence of aflatoxin‐associated HCC [69]. Addressing this burden requires strengthened food safety monitoring, climate‐adaptive agricultural practices, and targeted public health interventions.

The maximum allowable limits of aflatoxins in different countries and regions are presented in Table 5.

**Table 5:** Maximum allowable limits of aflatoxins in different countries.

Country/Organization	Type of food	Types of aflatoxins	Maximum (μg/kg)
Codex	Peanuts	Total aflatoxins	15
European Union	Peanuts	Aflatoxin B₁	8
		Total aflatoxins	15
	Peanut products	Total alfatoxins	4
		Aflatoxin B₁	2
FDA	Peanuts	Total aflatoxins	20
China	Peanut and corn	Aflatoxin B₁	20
India	All foods	Aflatoxin B₁	30
Indonesia	All foods	Total aflatoxins	35
	Peanuts and corn	Total aflatoxins	20
	Peanuts and corn	Aflatoxin B₁	15
Japan	All foods	Total aflatoxins	10
The Republic of Korea	Grains, cereal products	Aflatoxin B₁	10
Malaysia	Raw peanuts	Total aflatoxins	15
	Peanut products	Total aflatoxins	10
Philippines	All foods	Total aflatoxins	20
Singapore	All foods	Total aflatoxins	5
Sri Lanka	All foods	Total aflatoxins	30
Thailand	All foods	Total aflatoxins	20
Vietnam	All foods	Total aflatoxins	10

*Note:* The aflatoxin limit data for various countries/organizations in this table are all sourced from the official food safety regulatory texts issued by the respective countries, such as Commission Regulation (EC) No. 165/2010 (2010), US Food and Drug Administration (FDA), Codex Stan Cxs 193‐1995 (1995), Malasian Regulation Food Act 1983 (2014) and the National Food Safety Standard of China (GB 2761‐2017), among others.

### 5.4 Multifactorial Lifestyle Factors

HCC is closely associated with modifiable lifestyle risk factors, including excessive alcohol consumption, tobacco abuse, unhealthy dietary patterns, and physical inactivity. These behaviors contribute significantly to liver disease progression and carcinogenesis [70, 71]. Alcohol is the second most rapidly growing cause of HCC‐related deaths globally, following MASLD [15]. The annual incidence of alcohol‐related HCC ranges from 0.9% to 5.6% across diverse clinical cohorts [72]. A meta‐analysis involving 148,333 participants from 18 studies reported cumulative incidences of 1%, 3%, and 9% at 1, 5, and 10 years, respectively, among patients with alcohol‐associated cirrhosis [73]. The carcinogenic effects of alcohol are mediated by multiple mechanisms: (1) the production of acetaldehyde, a toxic metabolite; (2) increased oxidative stress driven by cytochrome P450 family 2 subfamily E member 1 (CYP2E1) and iron accumulation, compounded by impaired antioxidant defenses and DNA repair; (3) chronic inflammation resulting from immune dysregulation; and (4) disruption of methylation processes leading to epigenetic alterations [70, 71, 74]. Notably, the susceptibility to alcohol‐induced hepatocarcinogenesis exhibits marked interindividual variability, which is largely attributed to gene variations. The most renowned genetic variant is the aldehyde dehydrogenase 2 (*ALDH2*) rs671 polymorphism, which encodes a dysfunctional enzyme that impairs acetaldehyde metabolism and causes its accumulation in hepatocytes after alcohol intake. Carriers of the ALDH2*2 allele exhibit a significantly higher risk of alcohol‐associated cirrhosis and subsequent HCC, particularly in heavy drinkers, highlighting the critical role of genetic background in alcohol‐related HCC susceptibility [75, 76]. The ALDH2*2 allele exhibits a high prevalence in East Asian populations, which may also contribute to the high incidence of liver cancer in this ethnic group [77]. Strict alcohol abstinence is recommended for these patients [78]. Moreover, research shows that compared with individuals without HBV/HCV infection and no alcohol consumption history, the odds ratio (OR) for HCC is 13.5 in those with HBV monoinfection, 12.2 in those with HCV infection, and 1.42 in those with only an alcohol consumption history. For patients with HBV infection plus alcohol consumption, the OR is 14.56, while for those with concurrent HCV infection and alcohol consumption, it reaches 42.44—indicating that viral hepatitis, especially hepatitis C, exerts a synergistic effect with alcohol in promoting HCC [79]. According to the GBD database, the global ASIR of alcohol‐related liver cancer increased from 1.0 to 1.1 per 100,000 between 1990 and 2021, paralleled by a similar rise in the ASMR (Figure 8A,B). In China, the ASIR increased with fluctuations from 0.8 to 0.9 per 100,000, whereas the ASMR remained relatively stable (Figure 8C,D). An increasing body of evidence implicates cigarette smoking in exerting adverse effects on fatty liver disease. Approximately 20% of individuals with MASLD are current smokers [80, 81]. Tobacco smoke contains carcinogens that induce DNA damage, promote gene mutations, and enhance the risk of malignant transformation [82]. Furthermore, tobacco‐related toxins activate hepatic stellate cells (HSCs) via inflammatory cytokines, promoting fibrosis and cirrhosis, both of which predispose to HCC [83, 84]. Oxidative stress from smoking also contributes to hepatocellular damage and fibrogenesis [84]. Smoking demonstrates synergistic effects with chronic HBV and HCV infections, amplifying HCC risk [83, 84]. According to data from an epidemiological study in China, using the general population who are hepatitis B surface antigen (HBsAg)‐negative and non‐smokers as the reference group, the risk of developing HCC in individuals who are HBsAg‐positive but non‐smokers is 7.66 times that of the general population. In contrast, the risk of HCC in individuals who are HBsAg‐positive and have a smoking history reaches 15.68 times that of the general population [85]. It may also indirectly accelerate MASLD progression through mechanisms involving insulin resistance (IR), and it exhibits additive effects when combined with diabetes [86]. Evidence also suggests smoking interferes with HBV treatment by maintaining high viral loads [86]. Three large‐scale meta‐analyses confirm that smoking increases the risk of HCC, with current smokers demonstrating a significantly higher risk than former smokers [87, 88, 89]. Diet is a pivotal factor in HCC development, particularly through pathways involving obesity and IR [90, 91, 92, 93]. Diets high in sugar and fat, particularly from snacks and processed foods, are associated with greater hepatic fat accumulation [94]. High sugar intake promotes MASLD, with sugar‐sweetened beverages identified as particularly harmful [95]. A cohort study involving 477,206 participants found that consuming 50 g of total sugar daily increased HCC risk by 43% [96]. Conversely, healthy dietary patterns are protective. Increased vegetable intake is especially beneficial; for example, 10 g/day dietary fiber reduces HCC risk by 30% [96]. A meta‐analysis reported that each additional 100 g/day of vegetable intake was associated with an 8% lower risk of HCC, whereas fruit consumption did not confer the same benefit [97]. A high‐fiber diet may reduce cancer risk through multiple mechanisms, including attenuated insulin secretion and IR, decreased growth factor activity, anti‐inflammatory effects from short‐chain fatty acids produced by gut microbiota, and reduced obesity risk [97]. The Mediterranean diet, rich in vegetables, fish, and fiber and low in sugar, has been associated with a lower HCC incidence due to its anti‐inflammatory properties [98]. Additionally, a higher intake of plant‐based fats is inversely associated with HCC incidence compared to animal‐based fats [99]. Sedentary behavior is increasingly recognized as an independent risk factor for MASLD and HCC [100]. Physical activity has been shown to reduce hepatic fat by 20%–30% independent of weight loss, highlighting its utility in preventing MASLD‐related liver cancer [101]. A meta‐analysis of 98 studies found that increasing physical activity from 0 to 4000 metabolic equivalent (MET) minutes per week reduced the risk of HCC by 2.7%, and further increasing activity levels to 13,200 MET‐minutes per week resulted in a 5.9% risk reduction [102].

**Figure 9:**
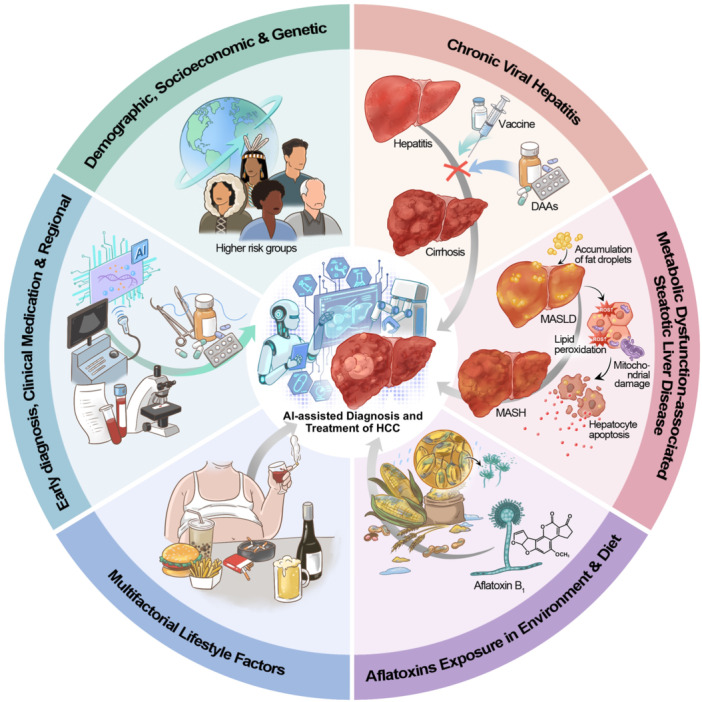
Schematic overview of key components and future directions in liver cancer prevention, diagnosis, and management. This illustration summarizes essential strategies across the continuum of liver cancer control, including risk factor mitigation, early detection, precision diagnostics, therapeutic advancements, and health policy initiatives. Future recommendations emphasize integrative, multidisciplinary approaches to reduce disease burden and improve patient outcomes globally. AI, artificial intelligence; DAAs, direct‐acting antivirals; HCC, hepatocellular carcinoma; MASH, metabolic dysfunction‐associated steatohepatitis; MASLD, metabolic dysfunction‐associated steatotic liver disease; ROS, reactive oxygen species.

### 5.5 Role of Routine Surveillance, Early Diagnosis, and Treatment

Due to the insidious nature of symptom onset, more than 50% of HCC cases are diagnosed at an advanced stage [103], which significantly worsens prognosis and contributes to a high mortality rate. Guidelines from the American Association for the Study of Liver Diseases (AASLD), the European Association for the Study of the Liver (EASL), and the Asian Pacific Association for the Study of the Liver (APASL) recommend semi‐annual ultrasound‐based surveillance for high‐risk individuals [104, 105, 106]. A randomized controlled trial conducted in China, which enrolled patients with HBV infection or a history of chronic hepatitis, demonstrated that combining semi‐annual alpha‐fetoprotein (AFP) testing with ultrasound substantially improved early HCC detection. Subclinical or small tumors were detected in 60.5% and 45.3% of cases, respectively. This approach was associated with a significantly higher rate of curative tumor resection (46.5% vs. 7.5%) and reduced HCC‐related mortality (hazard ratio [HR] 0.63, 95% CI: 0.4–1.0) [107]. Surgical interventions, including hepatic resection and liver transplantation, remain the primary curative options for HCC [108]. However, only 30%–40% of patients are suitable candidates for these procedures, primarily due to delayed diagnosis [109, 110]. The remaining 60%–70% of patients with unresectable tumors require systemic therapies, often in combination with locoregional treatments such as radiofrequency ablation, microwave ablation, or transcatheter arterial chemoembolization [111], as well as radiotherapy [112]. Several systemic agents have shown efficacy for unresectable HCC. These include multi‐target tyrosine kinase inhibitors (TKIs) such as sorafenib, lenvatinib, and donafenib [113]; programmed cell death protein 1 (PD‐1) blockade immunotherapy with tislelizumab [114]; and antiangiogenic agents such as bevacizumab [115]. Combination regimens have demonstrated enhanced efficacy over monotherapies. Notable phase III clinical trials include IMbrave 150 (atezolizumba plus bevacizumab) [116], ORIENT‐32 (sintilimab plus a bevacizumab biosimilar) [117], CARES‐310 (camrelizumab plus rivoceranib) [118], HIMALAYA (durvalumab plus tremelimumab) [119], and CheckMate‐9DW (nivolumab plus ipilimumab) [120], all of which reported improved clinical outcomes in patients with unresectable HCC. Recently, our team reported results from the phase III CARES‐009 trial, which demonstrated that perioperative administration of camrelizumab plus rivoceranib significantly improved event‐free survival compared with surgery alone in patients with resectable HCC at intermediate or high risk of recurrence (median event‐free survival: 42.1 vs. 19.4 months; HR = 0.59, *p* = 0.004) [121]. Despite curative resection, the recurrence rate of HCC can reach up to 80% [108, 121]. Even among patients meeting stringent liver transplantation criteria, 6%–18% may experience recurrence post‐transplantation [122, 123]. A meta‐analysis showed that the relative risk (RR) of 1.8 (95% CI: 1.7–2.0). The early detection rate in follow‐up groups was 58.2% (95% CI: 57.1%–59.3%), compared to 34.0% (95% CI: 33.1%–34.9%) in non‐follow‐up groups. Subgroup analyses across regions and different surveillance modalities (ultrasound alone or in combination with AFP) consistently demonstrated improved early detection and extended survival (HR = 0.6, 95% CI: 0.6–0.7) in patients receiving routine follow‐up [124]. Recurrence is particularly common in cases with macroscopic or microscopic vascular invasion and poor tumor differentiation [125]. In line with previous research, post‐curative treatment surveillance should include imaging (magnetic resonance imaging [MRI] or contrast‐enhanced computed tomography (CT)) every 3–6 months during the first 2 years. For patients with high‐risk features, such as tumors larger than 5 cm, microvascular invasion, elevated AFP levels, or persistent HBV viremia, the interval should be shortened to every 2–3 months. Between 3 and 5 years post‐treatment, imaging is recommended every 4–6 months. Beyond 5 years, the interval may be extended to every 6–12 months, depending on patient risk.

### 5.6 Demographic Factors Influencing Liver Cancer Epidemiology

Demographic variables are critical exposure factors in epidemiological investigations of liver cancer. These factors indirectly or directly modulate the risk of HCC by influencing hepatocellular physiology, disease susceptibility, and individual health behaviors. The mechanisms through which these demographic elements exert their effects vary in strength and complexity. Age is an inherent, non‐modifiable demographic factor. According to estimates from the World Health Organization (WHO), more than 2 billion individuals will be aged 60 years or older by 2050, and this demographic shift is expected to be accompanied by a greater burden of chronic diseases. Aging is associated with a heightened incidence of cancer [126] in general and is an independent risk factor for HCC [127]. It also contributes to an increased prevalence of HCC‐related conditions such as MASH and MASLD [128]. Furthermore, older adults with HBV infection [129] tend to have poorer prognoses, suggesting that aging contributes to HCC pathogenesis through both physiological deterioration and increased disease susceptibility. Sex is another fixed demographic factor that significantly influences HCC across epidemiological, clinical, and prognostic dimensions. Epidemiologically, males are two to three times more likely than females to develop HCC [3]. The average age at diagnosis is also earlier in males (59.2 years) compared to females (62.5 years) [130]. Clinically, female patients often present with tumors that are less invasive, more likely to be encapsulated, and less frequently multifocal or associated with major vascular invasion and metastasis. They are also more likely to be diagnosed at an early stage of disease and during the compensated phase of liver dysfunction. In terms of prognosis, females generally exhibit better outcomes, with a median disease‐free survival of 19.5 months, substantially longer than the 4.5 months observed in males. However, these sex‐related differences in survival tend to diminish after age 65, possibly due to the decline in estrogen levels post‐menopause, which may otherwise exert a protective effect against liver cancer [131]. Race and ethnicity are also important determinants of HCC risk. Substantial evidence links racial and ethnic background with differences in the prevalence of chronic viral hepatitis and liver cancer [132]. In the United States (US), HCC incidence has significantly declined across most racial and ethnic groups between 2015 and 2018 (APC: −5.6%), except for Black individuals (APC: −0.7%, not statistically significant) and American Indian/Alaska Native (AI/AN) populations, who experienced a significant increase (APC: +4.8%). Notably, AI/AN individuals and US‐born Hispanic persons have the highest incidence of HCC in the country [133]. Mortality rates remain disproportionately high among Black populations, largely due to delays in diagnosis and presentation at more advanced disease stages [134, 135, 136]. The elevated incidence of liver cancer in AI/AN and the Black populations is primarily driven by higher rates of hepatitis B and C infection [137], often linked to intravenous drug use [138, 139], limited healthcare access, and socioeconomic disadvantage. Geographic isolation and poverty further exacerbate these disparities by restricting access to screening, diagnosis, and treatment [140]. Among Hispanic populations, the high incidence of HCC is largely attributable to metabolic liver disease. Both MASLD and MASH are more prevalent in Hispanic individuals, who exhibit a significantly elevated risk of MASLD compared to White individuals (RR: 1.36; 95% CI: 1.08–1.73), with the highest proportion of MASH observed among all groups (RR 1.24; 95% CI: 1.02–1.52) [140]. Multiple factors contribute to this increased vulnerability. The Patatin‐like phospholipase domain‐containing 3 (PNPLA3) genetic variant, which is strongly associated with hepatic steatosis, is more prevalent among Hispanic individuals [141]. Additionally, unhealthy dietary patterns and lower levels of physical activity are more commonly reported in this group [142]. Socioeconomic barriers, such as lower insurance coverage, further delay diagnosis and restrict access to timely treatment interventions, compounding disease burden [143].

## 6 Future Directions and Recommendations

This comprehensive review synthesizes current global and regional trends in liver cancer incidence and mortality, elucidating the complex interplay between viral infections, metabolic dysfunction, environmental toxins, lifestyle behaviors, and demographic determinants. Data from GLOBOCAN 2022, the GBD Study 1990–2021, and multiple epidemiological investigations provide a robust evidence base for understanding the evolving epidemiology of liver cancer. These insights are critical for informing the development of precision public health strategies aimed at prevention, early detection, and improved management of HCC.

### 6.1 Prevention and Treatment of Viral Hepatitis

The most effective strategy for eliminating HBV‐related liver cancer is the prevention of HBV infection through immunization. HBV prevention encompasses both active immunization using the HBV vaccine and passive immunization via hepatitis B immunoglobulin (HBIG) [144]. The HBV vaccine, the first human vaccine developed using viral antigens derived from infected individuals, has been demonstrated to be safe and effective. Since 2009, the WHO has recommended universal HBV vaccination for all newborns as a critical measure to prevent mother‐to‐child (vertical) transmission and reduce the burden of HBV‐related HCC [144, 145]. Modeling studies based on aggregated clinical trials and cohort data have shown that achieving 90% global coverage of the full childhood vaccination schedule, even when excluding the birth dose, could prevent up to 75% of HBV‐related deaths. Moreover, administering the first vaccine dose within 24 h of birth further enhances the protective effect [146]. A large‐scale cohort study also demonstrated that HBV vaccination reduced the risk of HCC by 84% in vaccinated populations compared with those unvaccinated [147]. HBIG, a purified immunoglobulin G derived from human plasma, has been clinically validated for its efficacy in blocking vertical HBV transmission and providing post‐exposure prophylaxis [148]. It is estimated that nearly 50% of HBV infections are acquired through vertical transmission [149]. In cases where the maternal HBV viral load is high (≥ 20,000 IU/mL), newborns should receive both HBIG and a complete course of HBV vaccination to achieve effective and comprehensive protection [148, 150]. Unlike HBV, no vaccine has yet been developed for HCV [151]. Therefore, DAAs remain the cornerstone of treatment for chronic HCV infection due to their high efficacy, favorable safety profile, and cost‐effectiveness. The standard simplified treatment protocol recommends that treatment‐naive patients without cirrhosis receive either 12 weeks of sofosbuvir/velpatasvir (SOL/VEL) therapy or 8 weeks of glecaprevir/pibrentasvir (G/P) therapy. For patients with compensated cirrhosis, the duration of G/P treatment should be extended to 12 weeks. Treatment regimens should be further tailored based on HCV genotype; for example, grazoprevir/elbasvir is specifically indicated for genotype 1b infection [152, 153]. DAA therapy offers clinical benefit across all stages of HCV infection and can achieve a sustained virologic response (SVR) in more than 95% of patients across all HCV genotypes [154, 155]. A meta‐analysis further demonstrated that, among patients with HCV‐related cirrhosis, DAA treatment significantly reduced the risk of HCC by approximately 70%, suggesting its potential as a preventive intervention [156].

### 6.2 Reduction of Aflatoxins Exposure

A cost‐effective and user‐friendly method for detecting aflatoxin concentrations in food or feed is essential in regions with a high prevalence of contamination. Traditional detection techniques include chromatographic methods and immunochemical assays such as enzyme‐linked immunosorbent assay (ELISA) or immunochromatographic assay (ICA), as well as immunosensor technologies [157]. Among these, portable immunosensor devices have shown promise for on‐site aflatoxin screening. For example, one study conducted in Uganda evaluated a handheld immunosensor for detecting aflatoxins in maize flour collected from six markets and multiple households. The results were consistent with those obtained using the standard ELISA method, demonstrating comparable accuracy and potential for field deployment [158]. In addition, polymerase chain reaction (PCR) technology has been increasingly utilized for the rapid detection of aflatoxins. PCR can amplify species‐specific genes, as well as regulatory and structural genes associated with aflatoxin biosynthesis, allowing for rapid, high‐throughput screening [159]. A study in South Africa demonstrated the efficacy of PCR‐based detection in identifying aflatoxins in animal feed, highlighting its potential applicability in resource‐limited settings [160]. Beyond detection, stringent regulatory frameworks are required to prevent aflatoxin‐contaminated food and feed from entering the supply chain. High‐income food‐producing countries typically possess the economic and institutional resources necessary to enforce such regulations effectively. In contrast, populations in low‐ and middle‐income countries often face significantly higher dietary exposure to aflatoxins due to the absence of regulatory mechanisms and enforcement capacity [161]. A 2003 report from the Food and Agriculture Organization (FAO) indicated that only 15 African countries had established aflatoxin‐specific regulatory standards. Many others lacked maximum residue limit legislation, largely due to insufficient data on toxin prevalence and limited technical capacity to conduct toxicological risk assessments or enforce compliance [66].

Finally, public health education plays a critical role in mitigating aflatoxin exposure. Raising consumer awareness of the health risks associated with aflatoxin‐contaminated food is essential for enabling informed decision‐making and fostering demand for safer, higher‐quality food products [162]. Educational campaigns targeting both producers and consumers can reinforce preventive measures across the food system.

### 6.3 Preventing the Progression of MASLD to MASH

Approximately 20%–30% of adults with MASLD will progress to MASH. Among those with MASH, around 20% may develop cirrhosis within a few years. Of patients with cirrhosis, approximately 45% will experience hepatic decompensation within the next decade, and 7% will develop HCC within 6.5 years [163]. Multiple strategies have been shown to reverse MASLD and prevent its progression to MASH and MASH‐related HCC. Weight loss remains the cornerstone of MASLD management. A reduction of as little as 5% in baseline body weight has been associated with decreased hepatic steatosis, while a reduction of 10% or more can lead to improvements in hepatic inflammation and fibrosis [164]. Beyond conventional physical activity, bariatric surgery offers additional benefits by enhancing endocrine function and promoting weight loss. Importantly, glucagon‐like peptide‐1 (GLP‐1) levels have been reported to increase after bariatric surgery even before substantial weight reduction occurs, suggesting endocrine modulation as a key mechanism [165, 166]. GLP‐1 receptor agonists such as semaglutide and liraglutide have demonstrated anti‐inflammatory effects and the capacity to reduce hepatic fat deposition in patients with MASH [167, 168]. Diet plays a pivotal role in both the development and progression of MASLD. Diets rich in refined carbohydrates, sugar‐sweetened beverages [169, 170], processed red meat [171], and saturated fats [172] are associated with an increased risk of MASLD. In contrast, increased consumption of coffee [173], poultry white meat [174], fatty fish rich in n‐3 polyunsaturated fatty acids [175], and plant‐based foods [176] has been linked to a reduced incidence. These dietary components exert their effects primarily through modulation of hepatic inflammation. In particular, chronic inflammation driven by sustained lipotoxicity plays a central role in the transition from MASLD to MASH and eventually to HCC [177]. Anti‐inflammatory dietary patterns may confer hepatoprotective effects and help to mitigate disease progression [178]. IR and type 2 diabetes mellitus (T2DM) are established independent risk factors for MASLD [179, 180]. T2DM at baseline is considered the strongest predictor of progression from MASH to cirrhosis and HCC and is positively correlated with all‐cause mortality [179]. As such, improving IR and controlling glycemic status are essential components of MASLD management [92]. Pharmacologic agents targeting IR and T2DM, such as GLP‐1 receptor agonists (semaglutide and liraglutide), glucose‐dependent insulinotropic polypeptide (GIP)/GLP‐1 dual receptor agonists (tirzepatide), and peroxisome proliferator‐activated receptor (PPAR) agonists (pioglitazone), have demonstrated efficacy in reducing hepatic fat, inducing MASH remission, and improving fibrosis. These effects are mediated through improvements in systemic and hepatic insulin sensitivity. Moreover, emerging weight loss‐independent therapies, such as fibroblast growth factor 21 analogs, have shown promise in improving MASLD by restoring insulin signaling and regulating lipid metabolism, further supporting the role of IR as a central mechanism in disease progression [92, 180]. Notably, patients with MASH‐HCC are frequently comorbid with obesity and type 2 diabetes. Therefore, the combined administration of metabolic disease‐targeted therapies (e.g., sodium‐glucose linked transporter 1/2 [SGLT1/2] inhibitors) and HCC immunotherapies (e.g., PD‐1/programmed death‐ligand 1 [PD‐L1] inhibitors) is considered a promising strategy for clinical application [181].

Furthermore, it is noteworthy that the *PNPLA3* gene, particularly the rs738409 C > G mutation, has been identified as a significant genetic modifier of HCC risk, demonstrating an exceptionally high associated risk in MASLD‐related HCC [182, 183]. For patients carrying this specific mutation, a small interfering RNA (siRNA)‐based *PNPLA3* treatment has entered Phase 1 trials [184], marking a pivotal step toward genotype‐directed management of MASLD and its malignant complications.

### 6.4 Lifestyle Intervention

Alcohol consumption and smoking are well‐established independent risk factors for HCC [185]. Moreover, these two behaviors act synergistically to promote hepatocarcinogenesis through mechanisms such as oxidative stress, genomic instability, and dysregulation of the gut–liver axis [186]. Daily alcohol intake is associated with an elevated risk of liver fibrosis and its progression to cirrhosis when compared with non‐drinkers [72]. Among individuals with alcohol use disorder (AUD), the RR of developing HCC is approximately 2.4; this risk increases substantially to 22.4 in those with coexisting cirrhosis [13]. As a result, alcohol abstinence is considered the first‐line therapeutic approach for patients with impaired liver function and cirrhosis [187]. It is widely recognized as the most effective strategy across all stages of alcoholic liver disease [188]. Initiating abstinence in the early stages can attenuate hepatic inflammation, restore immune surveillance, and interrupt pro‐carcinogenic pathways, thereby highlighting a critical therapeutic window before irreversible liver damage [189, 190, 191]. In patients with compensated alcoholic cirrhosis, abstinence reduces the cumulative incidence of HCC from 25.7% to 8.1%. However, once decompensated manifestations such as portal hypertension emerge, the protective effect of abstinence against HCC is markedly diminished [190]. In addition to individual‐level interventions, population‐based strategies have proven effective and economically viable. These include increasing alcohol excise taxes and implementing minimum unit pricing policies, both of which have demonstrated measurable reductions in overall alcohol consumption [192]. For individuals with persistent heavy drinking, routine liver disease screening is strongly recommended [193]. Early detection of liver abnormalities not only facilitates timely medical intervention but also serves as a behavioral catalyst that encourages reductions in alcohol intake. Evidence suggests that individuals with more advanced liver damage exhibit a greater propensity to modify harmful drinking behaviors due to heightened health awareness [193]. Therefore, a comprehensive approach integrating behavioral counseling, pharmacologic support, and structured surveillance is essential to reduce the long‐term burden of HCC in alcohol‐dependent populations. Smoking cessation also plays a vital role in reducing the risk of HCC. The risk of developing HCC declines progressively with the duration of smoking abstinence. Notably, individuals who have abstained from smoking for more than 30 years exhibit a risk profile similar to that of never‐smokers [88]. Furthermore, smoking cessation contributes to improved outcomes in patients with HCC by reducing the risk of recurrence following curative hepatic resection [194]. In conclusion, early and sustained alcohol abstinence, particularly before the onset of hepatic decompensation, represents a cornerstone strategy in the prevention of HCC among individuals with AUD and cirrhosis. When combined with timely surveillance, behavioral therapy, and public health interventions, this integrated approach offers the greatest potential to prevent both the development and recurrence of liver cancer.

### 6.5 Support in Regions under Resource Constraints

Regions with limited economic development and inadequate healthcare infrastructure, such as those in Africa, face significant challenges across the entire spectrum of liver cancer control. These challenges range from HBV vaccination and treatment to HCC screening and management. In many African nations, healthcare resources are disproportionately allocated to infectious diseases such as Human Immunodeficiency Virus (HIV) and malaria. As a result, funding for viral hepatitis prevention and control programs is often insufficient [195, 196].

One critical area of intervention is improving the coverage of HBV vaccination. In West Africa, for instance, the childhood HBV vaccination coverage rate was only 72% in 2022, which is well below the global target of 90%. More concerning is the low coverage of the birth dose, which is essential for preventing mother‐to‐child (vertical) transmission, and stood at just 10%. Among adults, the HBV vaccination rate in the WHO African Region was only 18% in 2023, compared to the global average of 45% [197]. Until 2021, HBV vaccines were not provided free of charge in many African countries [198], and individuals were often required to pay out of pocket. This financial burden has contributed to low vaccination adherence [196]. The Global Alliance for Vaccines and Immunizations (GAVI) has played a pivotal role in promoting HBV vaccination. Since 2002, GAVI has partnered with the Chinese government to provide free birth‐dose HBV vaccines and has achieved notable success [199]. A study conducted a lifelong simulation on a cohort of 10 million newborns born in China in 2002 to analyze the economic benefits of HBV vaccination for newborns in China, with vaccine resources supported by the GAVI. The results showed that newborn HBV vaccination could increase 743,000 life‐years and 620,000 quality‐adjusted life‐years (QALYs). Economically, at just US$3.6 per newborn, the vaccination saves over US$1.429 billion in medical costs and productivity losses from a societal perspective, and US$1.059 billion in direct medical costs from a healthcare payer perspective [200]. However, GAVI′s collaboration with African countries began only in 2021 and was temporarily disrupted due to the Coronavirus Disease 2019 (COVID‐19) [198]. Looking ahead, a renewed GAVI‐Africa partnership is planned for 2026 to 2030. This initiative is expected to expand vaccine access and improve immunization rates across the continent [198, 201]. Beyond vaccination, many regions cannot afford key diagnostic and treatment tools. HBV DNA testing, non‐invasive liver fibrosis assessments such as transient elastography, and even basic serological screening remain largely inaccessible due to high costs and limited availability [196]. Medications for HBV are also unaffordable in many settings and are subject to unstable supply chains. Compounding the problem, HCC screening is nearly nonexistent in these regions. Approximately 92% of liver cancer patients in Africa are diagnosed at advanced stages, with a 1‐year mortality rate approaching 100%. Published incidences of HCC in the Black population of sub‐Saharan Africa underestimate the true incidence of the tumor because of the many instances in which HCC is either not definitively diagnosed or is not recorded in a cancer registry. Despite this, it is manifestly evident that the tumor occurs commonly and is a major cause of cancer deaths in Black African peoples living in the sub‐continent, particularly in those living in rural areas. Forty‐six thousand new cases of HCC have been recorded to be diagnosed in sub‐Saharan Africa each year, and age‐standardized incidences of the tumor as high as 41.2/100,000 persons/year have been documented. The highest incidence of HCC has been recorded in Mozambique. The tumor occurs at a young age in rural dwelling and, to a lesser extent, urban dwelling Black Africans. It is also more common in men than in women, particularly in the younger patients. Cirrhosis co‐exists with HCC in about 60% of patients and is equally common in the two sexes. The tumor is not only common in the Black African population, it also carries an especially grave prognosis, with about 93% of the patients dying within 12 months of the onset of symptoms. Caucasians living in the sub‐continent have a low incidence of HCC and it occurs at an older age. To address these limitations, cost‐effective and user‐friendly diagnostic alternatives should be prioritized. Rapid test strips for HBsAg provide a viable solution for population‐level HBV screening. A study has evaluated two ICAs, Dainascreen HBsAg for detecting human hepatitis HBsAg and Dainascreen Ausab for detecting human hepatitis B surface antibody (anti‐HBs) in human serum. The ICA systems are composed of a comb‐shaped device that contains nitrocellulose strips on which complexes of HBsAg and anti‐HBs can be visualized. The results can be read within 15 min of incubation. The limit of detection for HBsAg was 3.1 ng/mL and that for anti‐HBs was 42 mIU/mL. Results of HBsAg detection agreed completely with those of conventional enzyme immunoassays (EIAs) and showed a 100% sensitivity (158 of 158 samples) and a 100% specificity (304 of 304 samples). The Dainascreen Ausab detected 184 of the 199 EIA‐positive samples (sensitivity, 92.5%) and yielded six positive results among the 281 EIA‐negative samples (specificity, 97.9%) [202]. Ensuring high sensitivity and specificity, the ICA is faster (15 min vs. 3 h) and more cost‐effective than EIA, with a cost only one‐fourth of that of EIA. No complex instrumentation is needed, making it recommended for routine use in clinical microbiology laboratories, especially in developing areas [202]. In addition, non‐invasive biomarkers for liver fibrosis, such as Fibrosis 4 Score (FIB‐4) (calculated as age × aspartate aminotransferase [AST]/[platelet × alanine aminotransferase {ALT}^0.5^]) and the gamma‐glutamyltransferase‐to‐platelet ratio (GPR) index can be implemented to identify individuals at risk of HBV‐related liver cirrhosis [196, 203, 204, 205]. A study showed that the combined screening with FIB‐4 and GPR costs only US$12.3 to identify one case of HBV‐related cirrhosis, which is significantly lower than the treatment cost for cirrhosis complications (US$18,000 per case) [206]. Notably, GPR index test costs less than US$3 per test, making it more economical than FIB‐4. Its cost‐accuracy ratio for identifying significant HBV‐related fibrosis (0.0375) outperforms that of FIB‐4 (0.0629). Particularly suitable as a screening method for hepatic fibrosis in underdeveloped regions, GPR test can reduce the missed diagnosis rate of cirrhosis in HBV patients by 42% and save over US$25,000 per patient in long‐term treatment costs for complications [205]. Finally, governments in resource‐limited regions should integrate liver cancer prevention and screening into national public health agendas. International funding mechanisms and technical support from global health organizations are essential to build sustainable infrastructure for liver cancer control [196].

### 6.6 Application of Artificial Intelligence (AI) in HCC Prevention and Management

Due to the substantial heterogeneity in risk factors and pathogenesis of HCC, current prediction and prognostic strategies remain limited in their accuracy and generalizability. In recent years, AI has emerged as a promising tool to enhance the comprehensive clinical management of HCC across various stages of the disease [207, 208].

In high‐risk populations, such as individuals with HBV, HCV, or MASLD, AI models that longitudinally integrate data from medication history, lifestyle factors, and laboratory results have enabled individualized HCC risk prediction and patient stratification for targeted surveillance [207, 209]. Multiple studies have demonstrated that machine learning algorithms outperform traditional logistic regression models. While conventional logistic models typically achieve area under the curve (AUC) values around 0.8, AI‐based models have reached AUCs close to 0.9, thereby providing greater diagnostic accuracy and stability (Table 6) [209, 210, 211, 212]. Effective risk stratification has practical implications. For example, a 5‐year follow‐up study found that only 1 out of 972 individuals categorized as low risk progressed to HCC [210]. AI also significantly enhances sensitivity and reproducibility in imaging‐based and pathological diagnoses. In the context of ultrasound screening, deep learning algorithms reduce operator dependence and attain diagnostic accuracy levels comparable to radiologists (~76%) and contrast‐enhanced CT (approximately 84.7%), with only a slight inferiority to MRI (Table 7) [213, 214, 215]. For CT and MRI interpretation, convolutional neural networks that integrate multiphase imaging data (e.g., triple‐phase CT) can distinguish HCC from benign hepatic lesions with accuracy rates near 84% and AUCs approaching 0.9. Furthermore, combining MRI portal venous‐phase data with CT imaging has been shown to improve discrimination of pathological subtypes, with AUCs around 0.8. This information can support surgical planning (Table 7) [216, 217, 218]. In digital pathology, AI systems trained on large datasets of annotated Hematoxylin and Eosin (HE) slides have achieved robust performance in lesion detection and tumor classification. The identification and definition of the invasive zone in solid tumors establish a robust two‐dimensional coordinate framework for the systematic characterization and AI‐driven assessment of tumor invasiveness and metastatic potential [5, 219]. Collaborative approaches that combine AI‐assisted assessment with pathologist review consistently outperform either method alone. Remarkably, image‐based AI can infer the expression of immune‐regulatory genes such as cytotoxic T‐lymphocyte‐associated protein 4 (CTLA4), inducible T‐cell co‐stimulator (ICOS), and absent in melanoma 2 (AIM2) directly from histopathological images, and these molecular predictions are associated with progression‐free survival in patients treated with atezolizumab plus bevacizumab (Table 8) [220, 221, 222]. For patients with advanced HCC undergoing systemic therapy, AI can integrate multi‐omic datasets, such as RNA‐seq, spatial transcriptomics, microRNA profiles, and DNA methylation, to identify disease‐specific molecular signatures and novel therapeutic targets. This capability enables a personalized approach to immunotherapy and targeted drug development (Table 9) [223, 224, 225, 226]. Additionally, dual‐modality deep learning models that combine preoperative clinical variables with CT imaging data can predict postoperative recurrence and mortality with AUCs ranging from 0.8 to 0.9. These models substantially outperform traditional predictors such as microvascular invasion and clinical risk scores, which typically show AUCs between 0.5 and 0.6 [227]. AI can also be employed to forecast post‐transplant recurrence by integrating clinical and biomarker data [228, 229], to predict survival outcomes after transarterial chemoembolization (TACE) based on imaging features [230], to predict the risk of postoperative mortality and avoid futile surgery [231], and to identify immunotherapeutic targets through genomic profiling. Collectively, these applications enhance prognostic precision and support the development of individualized follow‐up strategies [232].

**Table 6:** AI‐based prediction model for progression to HCC in CHB.

Model name	Application	AUC	AUC (conventional model)
PLAN‐B‐CURE [209]	CHB risk prediction	Training cohort (*n* = 944): 0.86 Internal validation cohort (*n* = 1102): 0.81–0.82 External validation cohort (*n* = 2741): 0.78–0.80	Training cohort (*n* = 944): 0.62–0.72 (*p* < 0.01) Internal validation cohort (*n* = 1102): 0.63–0.70 (*p* < 0.001) External validation cohort (*n* = 2741): 0.61–0.73 (*p* < 0.001)
ML‐HCC Prediction Model [210]	CHB HCC prediction	Training cohort (*n* = 960): 0.90–0.96	Training cohort (*n* = 960): 0.76–0.84 (*p* < 0.05)
		Validation cohort (*n* = 1937): 0.93–0.96	Validation cohort (*n* = 1937): 0.78–0.84 (*p* < 0.05)
DL‐RNN model [211]	HCV HCC prediction	Training cohort (*n* = 43,336): 0.75–0.77	Training cohort (*n* = 43,336): 0.68–0.69 (*p* < 0.05)
		Validation cohort (*n* = 4815): 0.76–0.81	Validation cohort (*n* = 4815): 0.67–0.71 (*p* < 0.05)
GRU‐RNN model [212]	HCV 3‐year HCC prediction	Training cohort (*n* = 442): 0.63–0.79	Training cohort (*n* = 442): 0.54–0.73 (*p* < 0.001)
		External validation cohort (*n* = 1050): 0.60–0.69	External validation cohort (*n* = 1050): 0.56–0.67 (*p* = 0.04)

Abbreviations: AI, artificial intelligence; AUC, area under the curve; CHB, chronic hepatitis B; DL‐RNN, deep learning‐recurrent neural network; GRU‐RNN, gated recurrent unit‐recurrent neural network; HCC, hepatocellular carcinoma; HCV, hepatitis C virus; ML‐HCC, machine learning‐hepatocellular carcinoma; PLAN‐B‐CURE, prediction of liver‐related outcomes using an artificial intelligence‐driven model for network‐after chronic hepatitis B functional cure.

**Table 7:** AI applications in focal liver lesion (FLL) diagnosis and surgical guidance.

Model name	Application	AUC	AUC (conventional model)
Model of lesion, background and clinic [99]	FLL diagnosis	Internal validation cohort (*n* = 369): 0.89–0.96	—
		External validation cohort (*n* = 328): 0.89–0.96	
FLL prognosis ensemble model [213]	FLL diagnosis	Internal validation cohort (*n* = 367): 0.86–0.97	Literature reported 0.72–0.74
		External validation cohort (*n* = 177): 0.85–0.88	
CNN‐based liver masses differentiation model [216]	Benign‐malignant differentiation of liver nodules in cirrhosis patients	Internal validation cohort (*n* = 142): 0.61–0.80	—
		External validation cohort (*n* = 36): 0.64–0.84	
Liver cancer differential SVM model [217]	Preoperative HCC and non‐HCC differentiation	CT‐based training cohort (*n* = 40) AUC not provided	Based on clinical experience (CT/MRT) 0.60–0.70
		CT‐based internal validation cohort (*n* = 13): 0.75–0.87	
		MRI‐based training cohort (*n* = 17) AUC not provided	
		MRI‐based internal validation cohort (*n* = 6): 0.80–1.0	

Abbreviations: AI, artificial intelligence; AUC, area under the curve; CNN, convolutional neural network; CT, computed tomography; FLL, focal liver leision; HCC, hepatocellular carcinoma; MRI, magnetic resonance imaging; SVM, support vector machine; “—”, not available.

**Table 8:** AI models for histopathological image analysis in liver cancer.

Model name	Application	AUC
CNN‐based HCC‐related gene mutation prediction model [220]	Predict the mutation of specific genes based on HCC H&E‐stained images	Internal validation cohort (*n* = 491): 0.903
DenseNet‐based pathological auxiliary classification model for liver cancer [221]	Distinguish primary liver cancer subtype	Internal validation cohort (*n* = 26): 0.71–0.96
		External validation cohort (*n* = 80): 0.81–0.88

Abbreviation: AI, artificial intelligence; AUC, area under the curve; CNN, convolutional neural network; H&E, Hematoxylin and Eosin; HCC, hepatocellular carcinoma.

**Table 9:** AI and omics‐based model for hepatocellular carcinoma prognosis.

Model name	Application	*C*‐index
Deep learning‐based multi‐omics integration model [223]	HCC prognosis	Training cohort (*n* = 216): 0.66–0.74
		Internal validation cohort (*n* = 144): 0.61–0.77
		External validation cohort (*n* = 1044): 0.67–0.82
Matrix stiffness‐related signature model [224]	HCC stratification and prognosis	External validation cohort (*n* = 1044): 0.66–0.67
Stepcox (forward) combined RSF [225]	HCC prognosis	0.84

Abbreviation: AI, artificial intelligence; HCC, hepatocellular carcinoma; RSF, random survival forest.

Unfortunately, AI‐assisted diagnosis and treatment of liver cancer requires stable internet connectivity, reliable power supply, and adequate hardware infrastructure, as well as accurate algorithms and trained personnel. Currently, its widespread adoption and application are limited by regional economic and technological development levels [233, 234].

## 7 Conclusion

Liver cancer is a multifactorial disease characterized by evolving etiologies that encompass viral, metabolic, environmental, lifestyle, and demographic influences. Although substantial progress has been made in the prevention of viral hepatitis and the development of systemic therapies, new challenges are emerging. These include the increasing burden of MASLD‐related HCC, environmental toxin exposures, and widening health disparities across regions. To address these complexities, future strategies must adopt a comprehensive, multi‐dimensional framework. Key components should include the widespread implementation of preventive vaccination programs, evidence‐based lifestyle interventions, and the promotion of early detection through accessible screening tools. Additionally, precision medicine approaches, such as genomics‐guided therapies and the integration of AI for risk stratification, are promising to advance individualized care for HCC patients, particularly in high‐resource settings with adequate medical and technological support. Equally important are policy‐level interventions that promote equitable healthcare delivery. Global efforts must prioritize collaborative partnerships and strategic resource allocation to ensure that prevention, diagnosis, and treatment are accessible across diverse healthcare settings. Only through coordinated, inclusive, and innovative approaches can the global burden of HCC be effectively reduced and patient outcomes significantly improved (Figure 9).

## Author Contributions

Conceptualization: Jian Zhou, Dan G. Duda, and Ao Huang. Project Administration: Jian Zhou, Ao Huang. Methodology: Jiayan Yan, Jiayi Wang, Jian Fan, Xinyi Cui, Yuxi Zhang, Xinrong Yang, Qiang Gao, Zhenbin Ding, Jia Fan and Zhaoyou Tang. Visualization: Jiayan Yan, Jiayi Wang, Jian Fan, Xinyi Cui, Jia Fan and Yuxi Zhang. Funding acquisition: Jian Zhou, Ao Huang, Jiayan Yan, and Xinrong Yang. Writing – original draft preparation: Jiayan Yan, Jiayi Wang. Writing – review and editing: Jian Zhou, Dan G. Duda, and Ao Huang. Final approval of manuscript: All authors.

## Conflicts of Interest

The authors declare no conflicts of interest.

## Ethics Statement

This review is based on secondary analysis of publicly available epidemiological data (GLOBOCAN 2022 and GBD 2021) and does not involve primary human or animal experiments. Therefore, ethical approval and informed consent are not required. All data analyses comply with the data usage policies of the original databases.

## Data Availability

All GLOBCAN 2022 data used in this article can be accessed via the website (https://gco.iarc.who.int/today/), and all data used in the GBD 2021 database are available through the website (https://gbd2021.healthdata.org/gbd-results/).
